# Supplementary material for: Three types of genes underlying the Gametophyte factor1 locus cause unilateral cross incompatibility in maize
Source: Nat Commun. 2022 Aug 3;13:4498. doi: 10.1038/s41467-022-32180-9 (PMC9349285; doi:10.1038/s41467-022-32180-9)
Supplement: Supplementary file 1 — Supplementary Information [file 41467_2022_32180_MOESM1_ESM.pdf]

Three types of genes underlying the *Gametophyte factor1* locus cause unilateral cross incompatibility in maize

Wang *et al.*

Supplementary Table 1. Confirmation of *ZmPRP3* genotype in 45 maize inbred lines from the AMP.

Accession	Phenotype	Genotype
GEMS63	*ga1*	*ZmPRP3*_Hap1
Gy386	*ga1*	*ZmPRP3*_Hap1
HTH-17	*ga1*	*ZmPRP3*_Hap1
B73	*ga1*	*ZmPRP3*_Hap1
GY386B	*ga1*	*ZmPRP3*_Hap2
CML223	*ga1*	*ZmPRP3*_Hap3
3H-2	*ga1*	*ZmPRP3*_Hap3
CIMBL102	*ga1*	*ZmPRP3*_Hap3
CIMBL71	*ga1*	*ZmPRP3*_Hap3
CIMBL89	*ga1*	*ZmPRP3*_Hap3
CML165	*ga1*	*ZmPRP3*_Hap3
Gy923	*ga1*	*ZmPRP3*_Hap3
H21	*ga1*	*ZmPRP3*_Hap3
Hua83-2	*ga1*	*ZmPRP3*_Hap3
05W002	*ga1*	*ZmPRP3*_Hap4
05WN230	*ga1*	*ZmPRP3*_Hap4
B11	*ga1*	*ZmPRP3*_Hap4
By809	*ga1*	*ZmPRP3*_Hap4
BZN	*ga1*	*ZmPRP3*_Hap4
CIMBL61	*ga1*	*ZmPRP3*_Hap4
CIMBL62	*ga1*	*ZmPRP3*_Hap4
CIMBL63	*ga1*	*ZmPRP3*_Hap4
CIMBL7	*ga1*	*ZmPRP3*_Hap4
CML229	*ga1*	*ZmPRP3*_Hap4
Gy798	*ga1*	*ZmPRP3*_Hap4
9782	*Ga1-M*	*ZmPRP3*_Hap2
CML166	*Ga1-M*	*ZmPRP3*_Hap2
04K5702	*Ga1-M*	*ZmPRP3*_Hap3
CML163	*Ga1-M*	*ZmPRP3*_Hap3
CML169	*Ga1-M*	*ZmPRP3*_Hap3
CML285	*Ga1-M*	*ZmPRP3*_Hap3
07KS4	*Ga1-M*	*ZmPRP3*_Hap4
CIMBL60	*Ga1-M*	*ZmPRP3*_Hap4
CIMBL65	*Ga1-M*	*ZmPRP3*_Hap4
CML130	*Ga1-M*	*ZmPRP3*_Hap4
CML170	*Ga1-M*	*ZmPRP3*_Hap4
CML226	*Ga1-M*	*ZmPRP3*_Hap4
CML286	*Ga1-M*	*ZmPRP3*_Hap4

Supplementary Table 2. Primers used for fine mapping of the *Ga1* locus and physical position in SK and B73 genomes

ID	Forward primer (5'-3')	Reverse primer (5'-3')	Physical position (B73)	Physical position (SK)
M1	CAAGGTTGTCCAAAGAACCAA	AAGCTGGTGGAGACTGGAGA	chr4:7940491	chr4:7802344
M2	CGAGAAAACCAAGCCACACG	ACACCGGCTCATGAGATTGA	chr4:8124153	chr4:7949866
M4	ATTCACGGAACGGAGACCTG	GGCAGATGGGTAACACGACT	chr4:8350201	chr4:8145440
M5	ATCTATCGCACAAGCCCTAA	TTGGCCCATGTCTTCTCA	chr4:9964029	chr4:8560382
M6	CGATGATGAAGATGACCCTT	AGCCAGCGTCCACAAATA	chr4:10208668	chr4:9956456
M7	GGTGGTTGTGATTTTCCTCGC	CATTTGGCGGGACAAAACGG	chr4:10444946	chr4:10036565
M8	TCTGACCAGCATCAGCACAG	GTTGCATTGCATGCCAGTCG	chr4:10502680	chr4:10091684
M9	ACCAGGAATAATCGTTCGCGT	CAGCAGCAGTTGTGCAGTTC	chr4:10619187	chr4:10290465
M10.1	GCCGTAGCAGGTCATGTTGT	ACGCTGCAATGATGGTCAAA	chr4:10884840	chr4:10575778

Supplementary Table 3. Primers used for re-sequencing, quantitative RT-PCR (qRT-PCR) analysis and transgenic experiments.

No.	ID	Forward primer (5'-3')	Reverse primer (5'-3')	Purpose
1	*ZmPME3*_qpcr	CTTCCGCGTCCTCAACAC	CGTAGTACAGTACGCCAGCA	qRT-PCR for *ZmPME3* in AMP and NIL
2	*ZmPRP3*_qpcr	CGGCAAGTGCATAAGGGTAT	TTGAGGTGGCCCATCTGGT	qRT-PCR for *ZmPRP3* in AMP and NIL
3	*PME*_qpcr	TGGCGGTTACACGACCATTAG	TCCTCGGACATTATGGTGATG	qRT-PCR for *ZmGa1Ps-m* in SK pollen
4	*ZmUbi*	GGCCGCACCTTAGCAGACTA	ATGGAGAGGGCACCAGACGA	Control for quantitative qRT-PCR
5	*ZmPME3*_OEqpcr	CTTCCGCGTCCTCAACAC	AGGTGGCATCGCCCTCGC	qRT-PCR for *ZmPME3* over-expression plants
6	*ZmPRP3*_target	GTGCAGGCAAAGAAAAGAGG	ACAGGAAGGATCTGGCTGAG	Re-sequence of *ZmPRP3 /*Sequencing editing target
7	*ZmGa1Ps-m*_OEqpcr	CCGAAGGTGGTCACGAGGGT	CCCTTTCTCGGGATACACTACA	qRT-PCR for *ZmGa1Ps-m* over-expression plants
8	*Zm00001d048948*_target	GAGAGTAGCTAGCGAAGC	CTTCTTGAACACCGTCTCGA	Re-sequence of *Zm00001d048948*/ Sequencing editing target
9	*Zm00001d048949*_target	CAGAAGCAAGTAGCAGCTACA	AGACGAGAGATCGATGCTGC	Re-sequence of *Zm00001d048949*/ Sequencing editing target
10	*Zm00001d048950*_target	AGGTCTCGCGCATGCATCTG	TACCTTACCCACTGCCAT	Re-sequence of *Zm00001d048950*/ Sequencing editing targett
11	*ZmPME3*_targe1	AGACGCGCACAAAGGTAAGT	TGTTGACGGTCTCGTCGTAG	Re-sequencing of *ZmPME3*
12	*ZmPME3*_targe2	CTACGACGAGACCGTCAACA	GTGCACATGTGGAACGAGAA	Re-sequencing of *ZmPME3*
13	*ZmPME3*_targe3	CACCGTGGACTTTGTGTTTG	ATAGGCTCAGGGACCCACTT	Re-sequencing of *ZmPME3*
14	*ZmPME3*_targe4	AAGTGGGTCCCTGAGCCTAT	AATCCATTTATTTAGATCGAGGATAC	Re-sequencing of *ZmPME3*

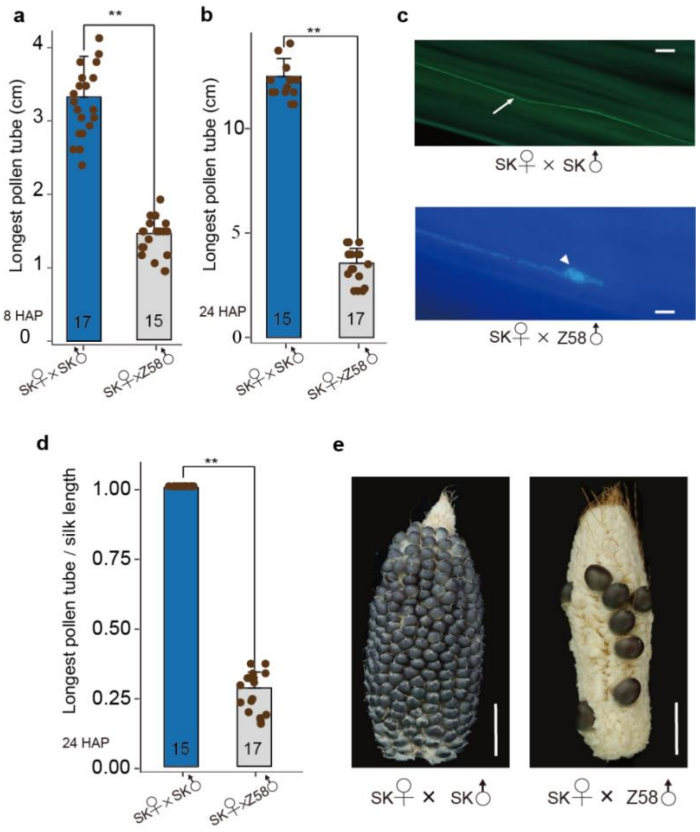

Supplementary Fig. 1. Seed set of crosses between SK and Z58. **(a)** Comparison of the longest pollen tube at 8 hours after pollination in silks of SK, using pollen of SK and Z58, respectively. **(b)** Comparison of the longest pollen tube at 24 hours after pollination in silks of SK, using pollen of SK and Z58, respectively. **(c)** Morphology of SK and Z58 pollen tubes in SK silks. Arrows point to normal SK pollen tubes, arrowhead indicates a heavy callose deposition at the tip of Z58 pollen tubes. Scale bar = 100μm. At least 15 pollen tubes were observed, and at least 5 pollen tubes showed the same morphological features. **(d)** Longest pollen tube as a percentage of the total silk length. **(e)** Crossing experiments showing ears of self-pollinated SK (*Ga1-S*) and crosses between the SK and Z58 (*ga1*) after SK silks were cut shorter than 5cm. Scale bar = 2 cm. For **a, b, d,** data were analysed by two-tailed Student' s t-test. Error bars represent mean + SD (**a, b, d**). Asterisks indicate significant differences (** P < 0.01) (**a, b, d**). Longest pollen tube length (n = number of biologically independent samples in each column) (**a, b, d**). Source data are provided as a Source Data file.

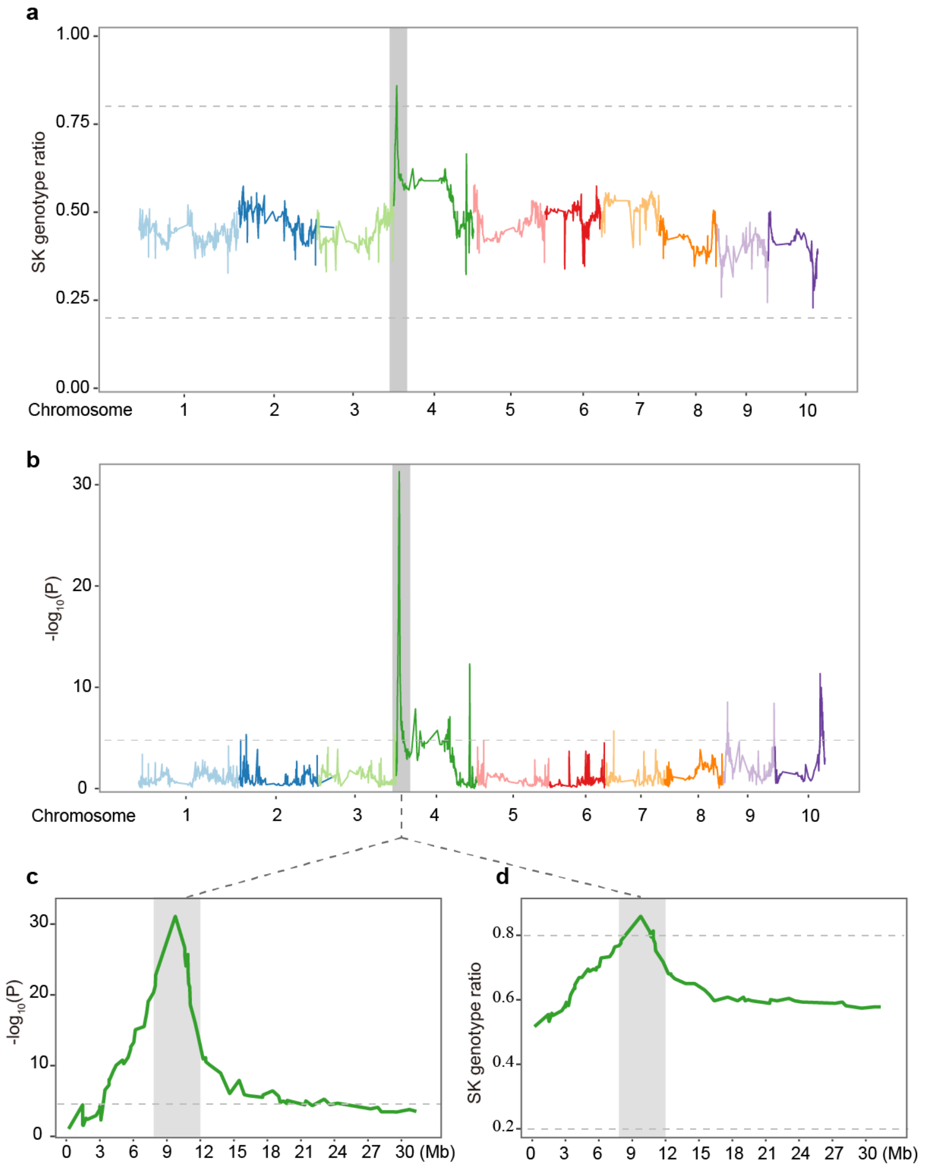

Supplementary Fig. 2. Segregation distortion locus detection along the ten chromosomes of the B73 genome. (a) Using the Bin-map of Z58/SK RIL, the ratio of alleles for each marker was counted [A (Z58); B (SK)], Setting A: B=1:1 as an expected value, and calculating percentage of B (SK) in each maker. Grey boxes represent the *Ga1* locus fine mapping region. **(b)** Performing the Chi-square Test to calculate -$\log_{10}$(P) in B73 genome. Counting the actual ratio of alleles for each marker [A (Z58); B (SK)] and setting A: B=1:1 as an expected value. **(c)** Performing the Chi-square Test to calculate -$\log_{10}$(P) on B73 chromosome4. **(d)** A segregation distortion locus on B73 chromosome4 that overlapped with the reported the *Ga1* locus (highlight with grey box).

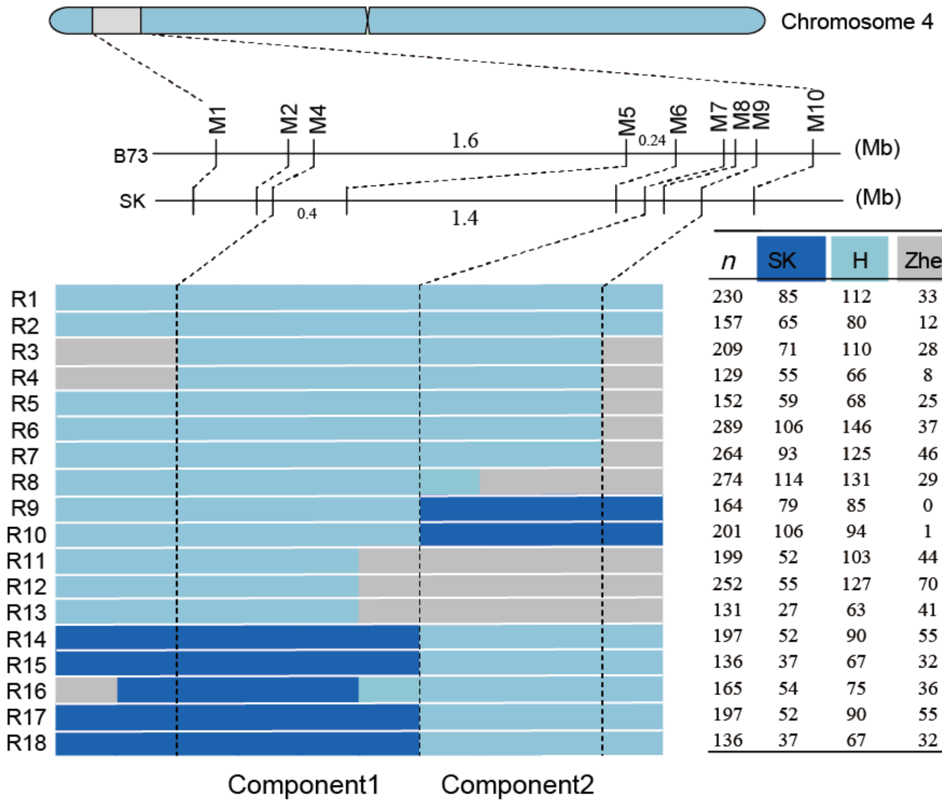

n	SK	H	Zheng58	P value
230	85	112	33	7.25E-06
157	65	80	12	1.65E-08
209	71	110	28	1.08E-04
129	55	66	8	3.53E-08
152	59	68	25	2.14E-04
289	106	146	37	6.90E-08
264	93	125	46	1.60E-04
274	114	131	29	2.72E-12
164	79	85	0	2.66E-17
201	106	94	1	9.91E-25
199	52	103	44	0.64
252	55	127	70	0.41
131	27	63	41	0.21
197	52	90	55	0.46
136	37	67	32	0.82
165	54	75	36	0.07
197	52	90	55	0.46
136	37	67	32	0.82

Supplementary Fig. 3. Fine mapping of the *Ga1* locus. Physical mapping showing that the *Ga1* locus was mapped to a region between marker M4 and M9, Component1 was mapped between marker M4 and M7 (R14, R15, R16, R17 and R18). Component2 was mapped between marker M7 and M9. (R9, R10, R11, R12 and R13). SK represents the SK genotype, Z58 represents Zheng58 genotype and H represents heterozygous genotype. The numbers in each row represent the number of individuals in the progeny generated after self-fertilization of recombinant plants with the genotype on the left. P-value represents the significance of the chi-square test.

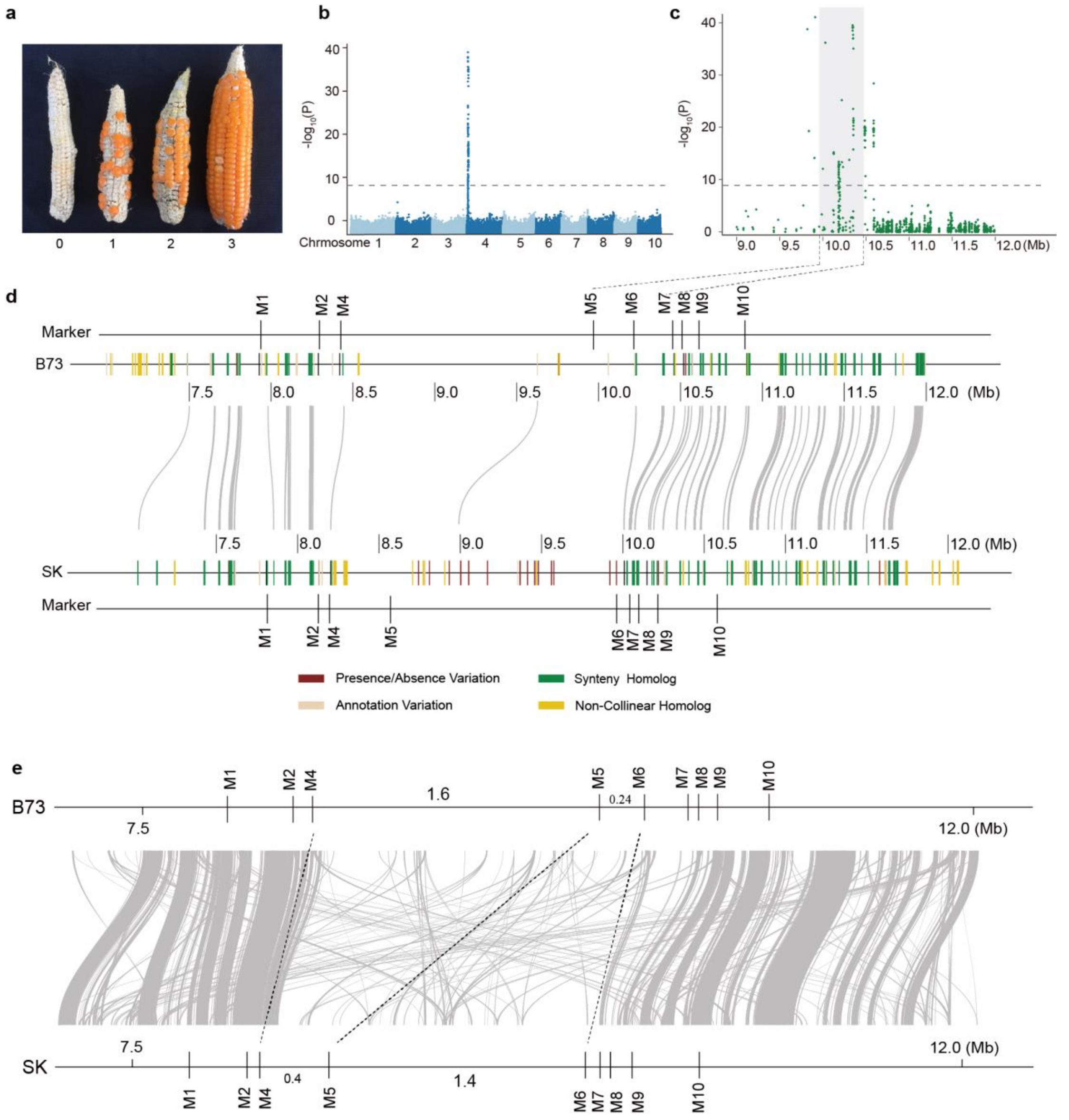

Supplementary Fig. 4. Component1 contains genes that determine pollen tube capability to overcome *Ga1-S* barrier. **(a)** The phenotype was assigned on a 0 to 3 score according to the ratio of seed set to present the degree of cross incompatibility and compatibility, when CML304 (*Ga1-S*) was used as the female parent and association mapping panel inbred lines were used as the male parent. **(b)** Genome-wide association analysis of male determinant when CML304 (*Ga1-S*) was used as the female parent and association mapping panel inbred lines were used as the male parent. Horizontal dashed line depicts the Bonferroni-adjusted significance threshold ($P=8\times10^{-9}$). **(c)** Genome-wide association signals located on chromosome4 in a 10~10.5 Mb interval in B73 genome. **(d)** Variation of SK (*Ga1-S*) and B73 (*ga1*) annotated genes were divided into four types: (1) homologous gene was detected in both B73 and SK genomes. (2) Presence/Absence Variation: gene

sequence presents in B73 genome but entirely absent from the whole SK genome or present in SK genome but absent from whole B73 genome. (3) Non-Collinear Homolog: homologous gene was detected in both B73 and SK genome, but are not located in the colinear region. (4) Annotation Variation: homologous gene sequence was detected in both B73 and SK genome, but one of them was not annotated as a functional gene in either B73 or SK genome. **(e)** Sequence alignment of the *Ga1* locus between B73 and SK genomes. Synteny blocks are highlighted by grey lines, dotted black lines indicated the variation of genomic size between M4 and M5, M5 and M6 in SK and B73 genomes, respectively. Source data are provided as a Source Data file.

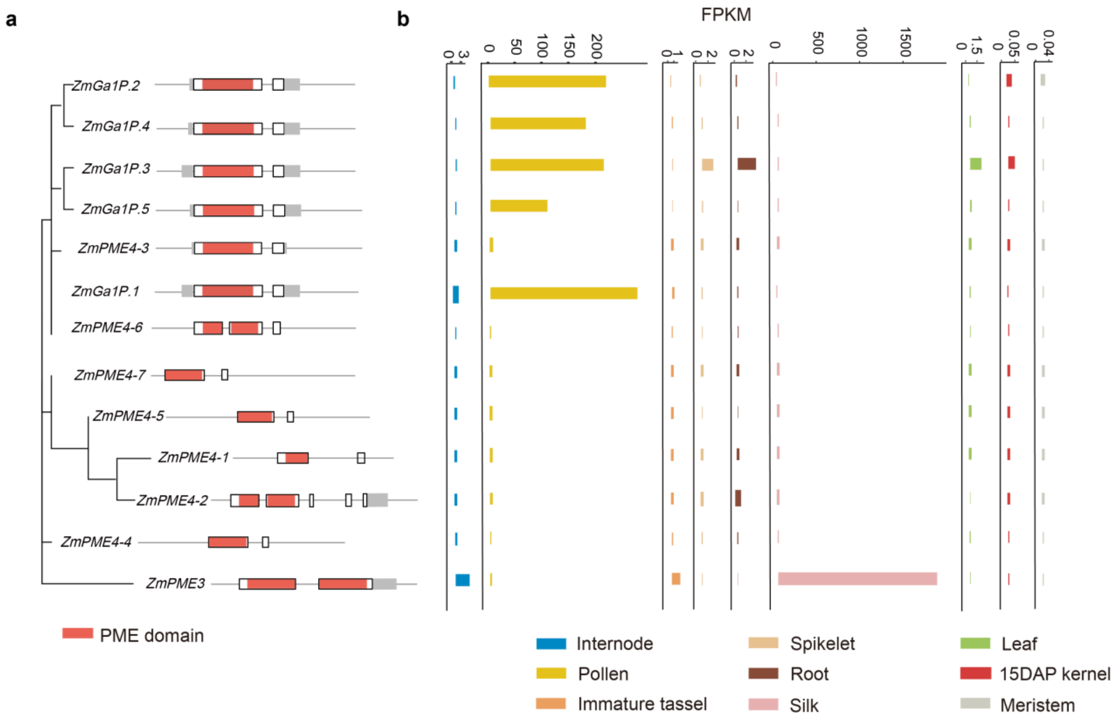

Supplementary Fig. 5. Gene structure of thirteen *PME* genes of maize and expression levels in nine SK tissues. (a) Gene structure of thirteen *PME* genes. White boxes represent coding sequences. The *PME* domain are highlighted with red boxes, and UTR regions are highlighted with grey boxes. **(b)** Expression levels of thirteen *PME* genes in nine SK tissues. FPKM, Fragments Per Kilobase per Million mapped reads.

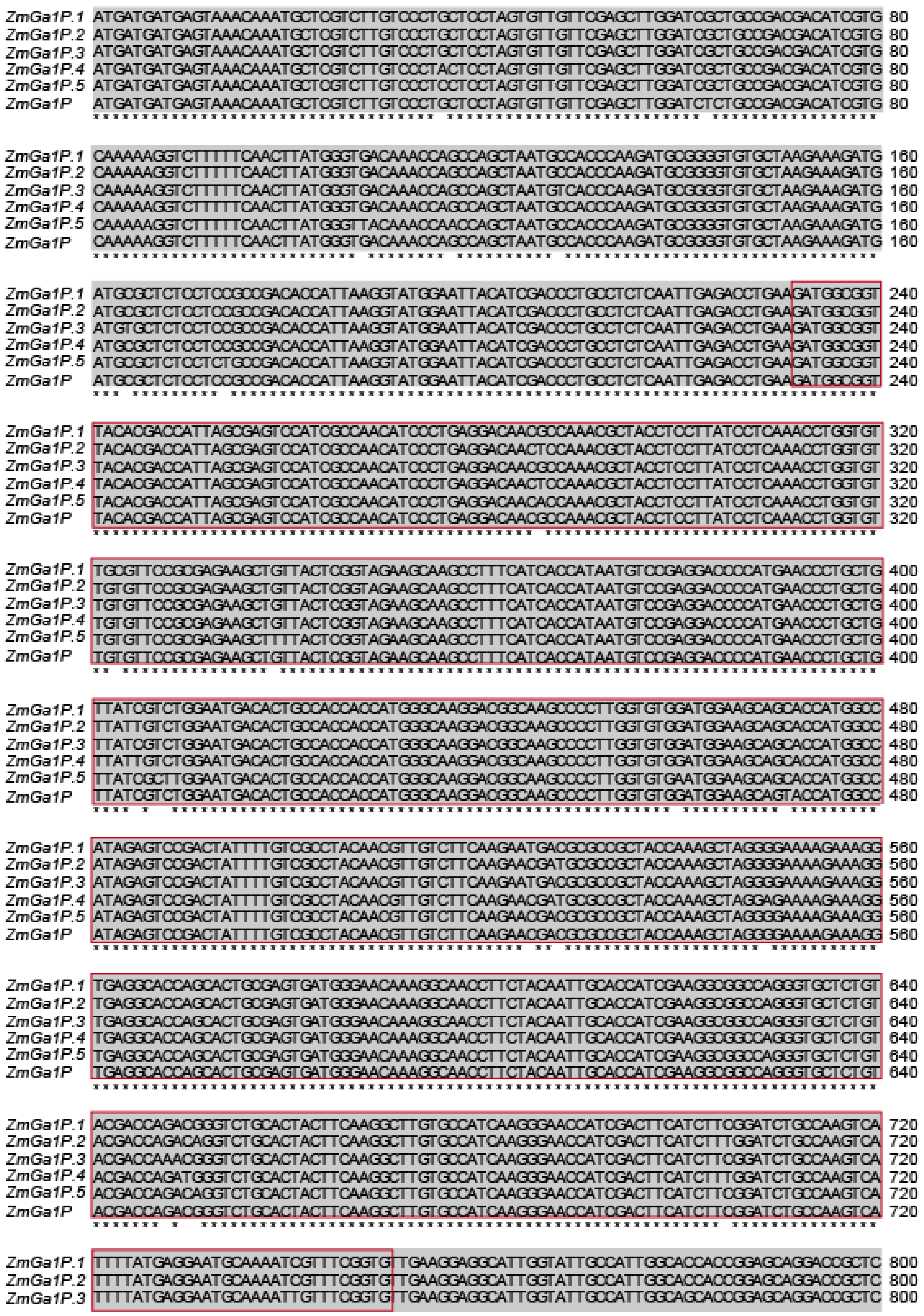
ZmGa1P.1
ZmGa1P.2
ZmGa1P.3
ZmGa1P.4
ZmGa1P.5
ZmGa1P
80
160
240
320
400
480
560
640
720
800

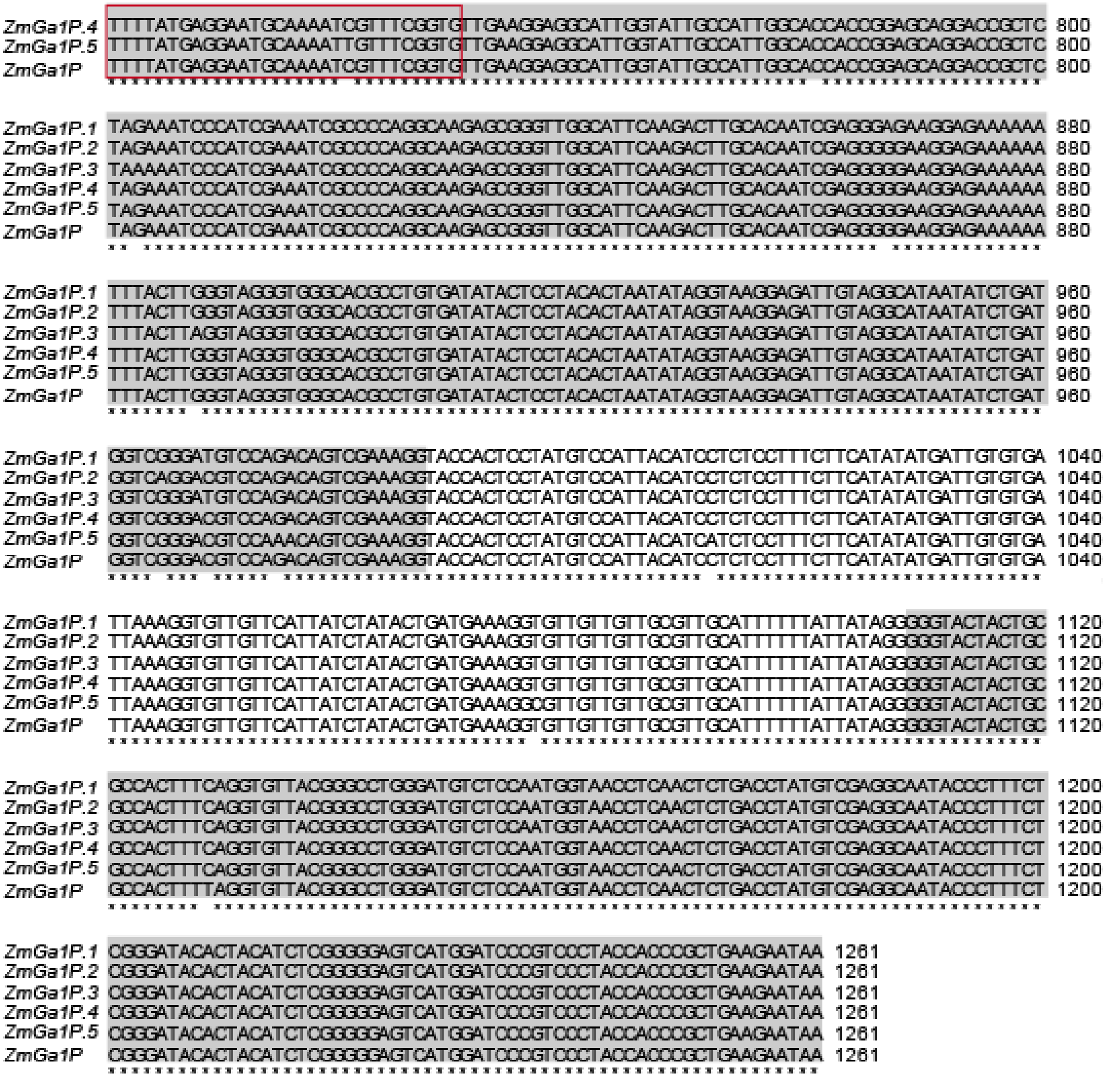

Supplementary Fig. 6. Genomic sequence alignment of *ZmGa1Ps-m* genes and *ZmGa1P*. “*” indicates identical nucleotides. The two exons in *ZmGa1Ps-m* are highlighted in grey. The *PME* domain is highlighted in red box.

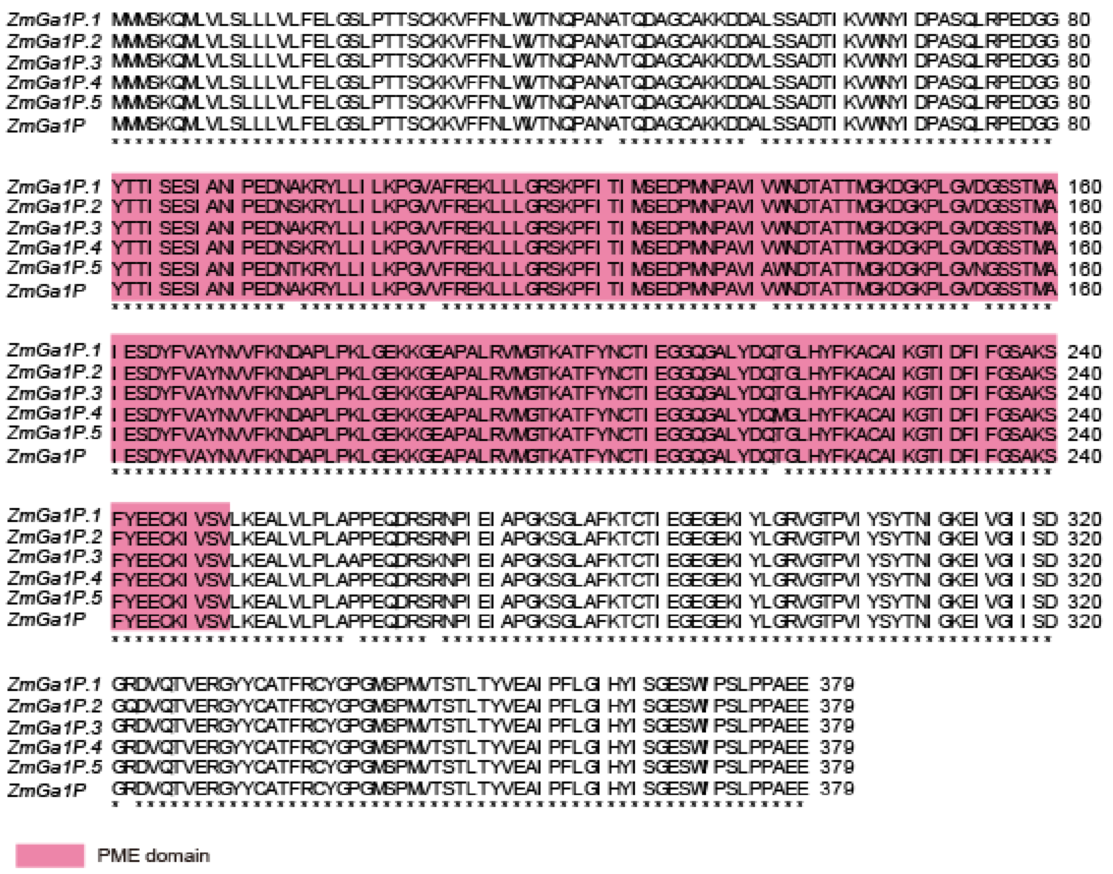

Supplementary Fig. 7. Protein sequence alignment of *ZmGa1Ps-m* genes and *ZmGa1P*. "*" indicates identical amino acids. The *PME* domain is highlighted in red.

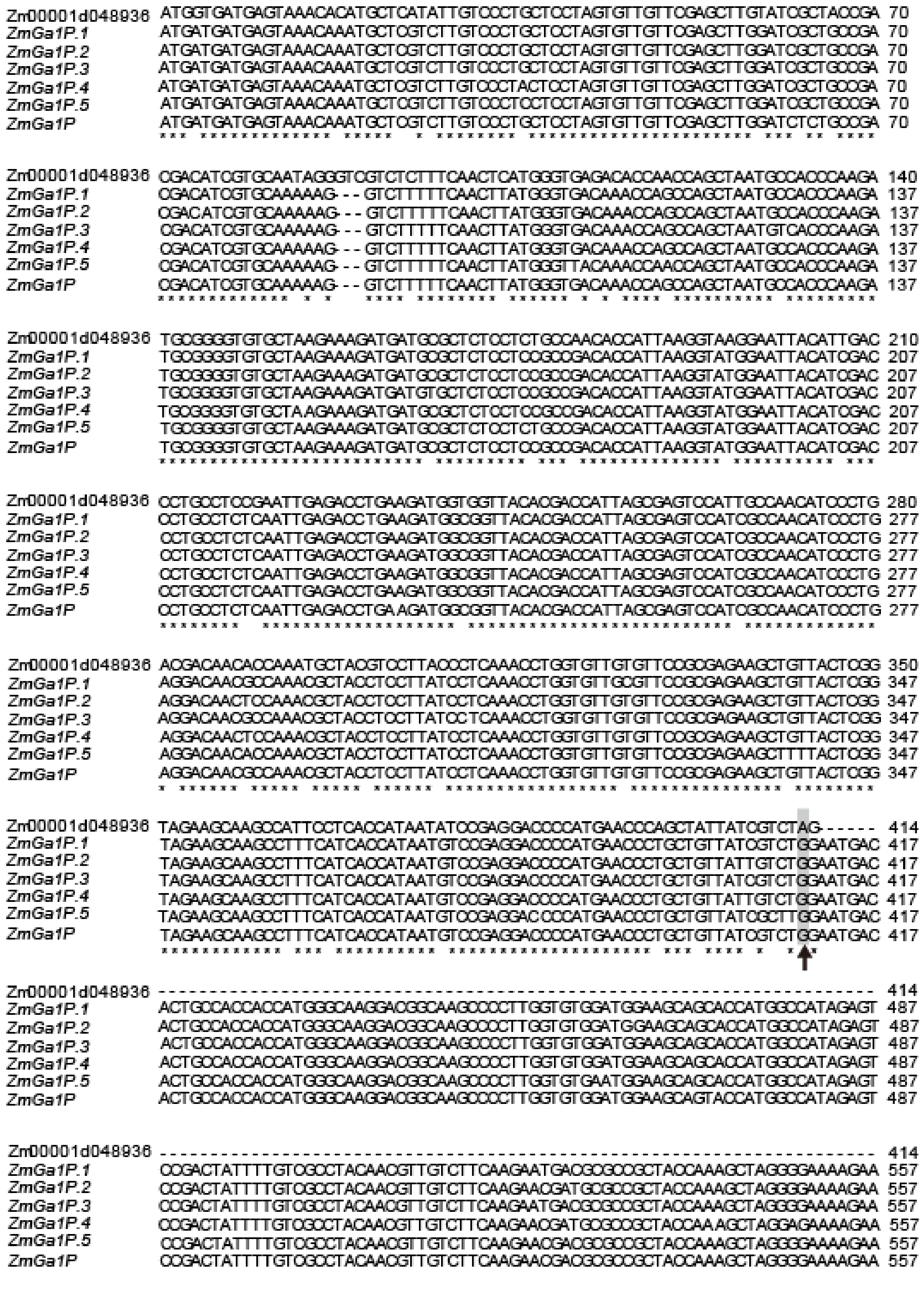

Supplementary Fig. 8. Genomic sequence alignment of *ZmGa1Ps-m, ZmGa1P* and *Zm00001d048936.* Arrow indicates the nonsense point mutation, and “*” indicates identical nucleotides.

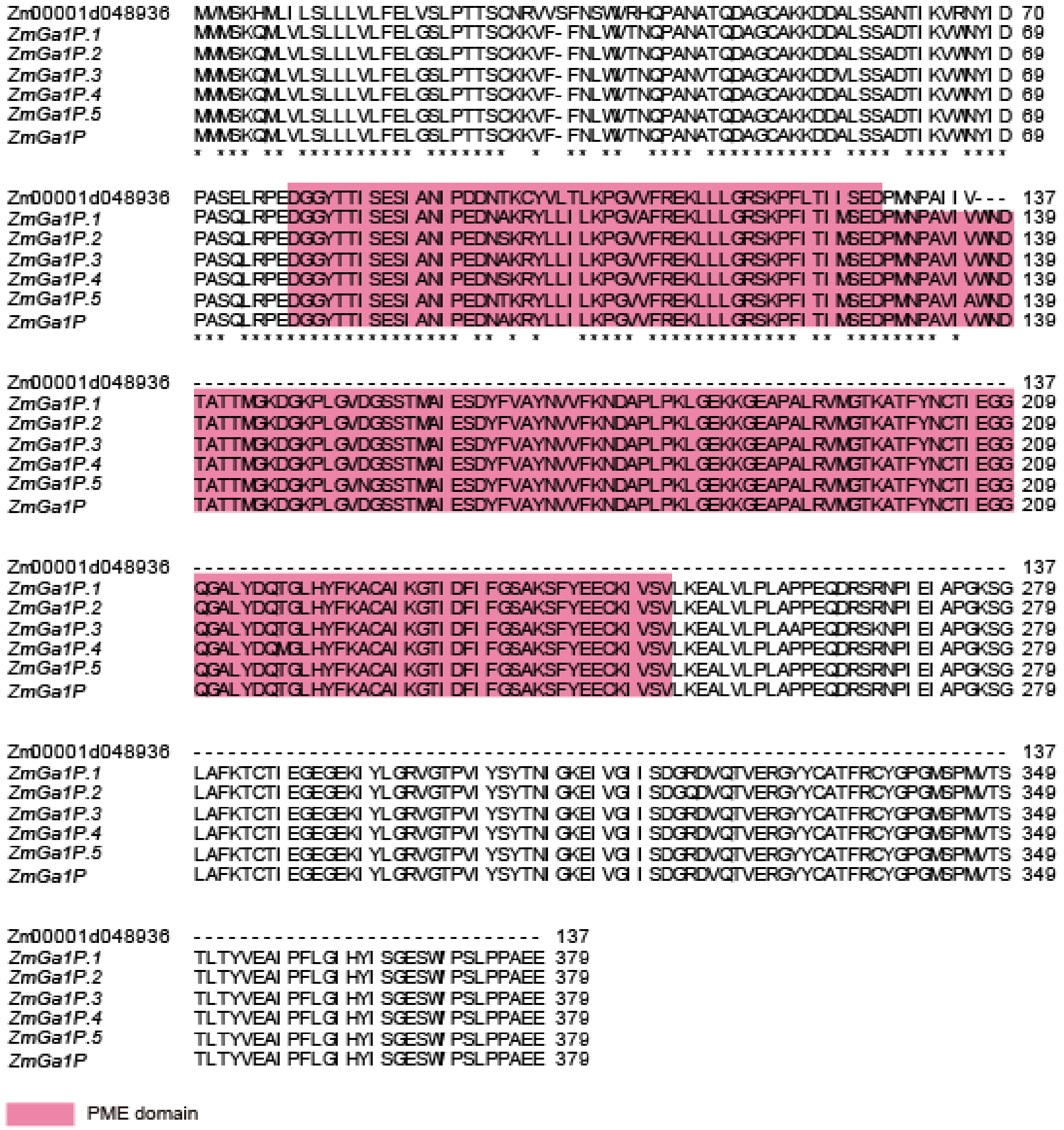

Supplementary Fig. 9. Sequence alignment of predicted proteins of *ZmGa1Ps-m, ZmGa1P* and *Zm00001d048936.* "*" indicates identical amino acids, and the *PME* domain is highlighted in red.

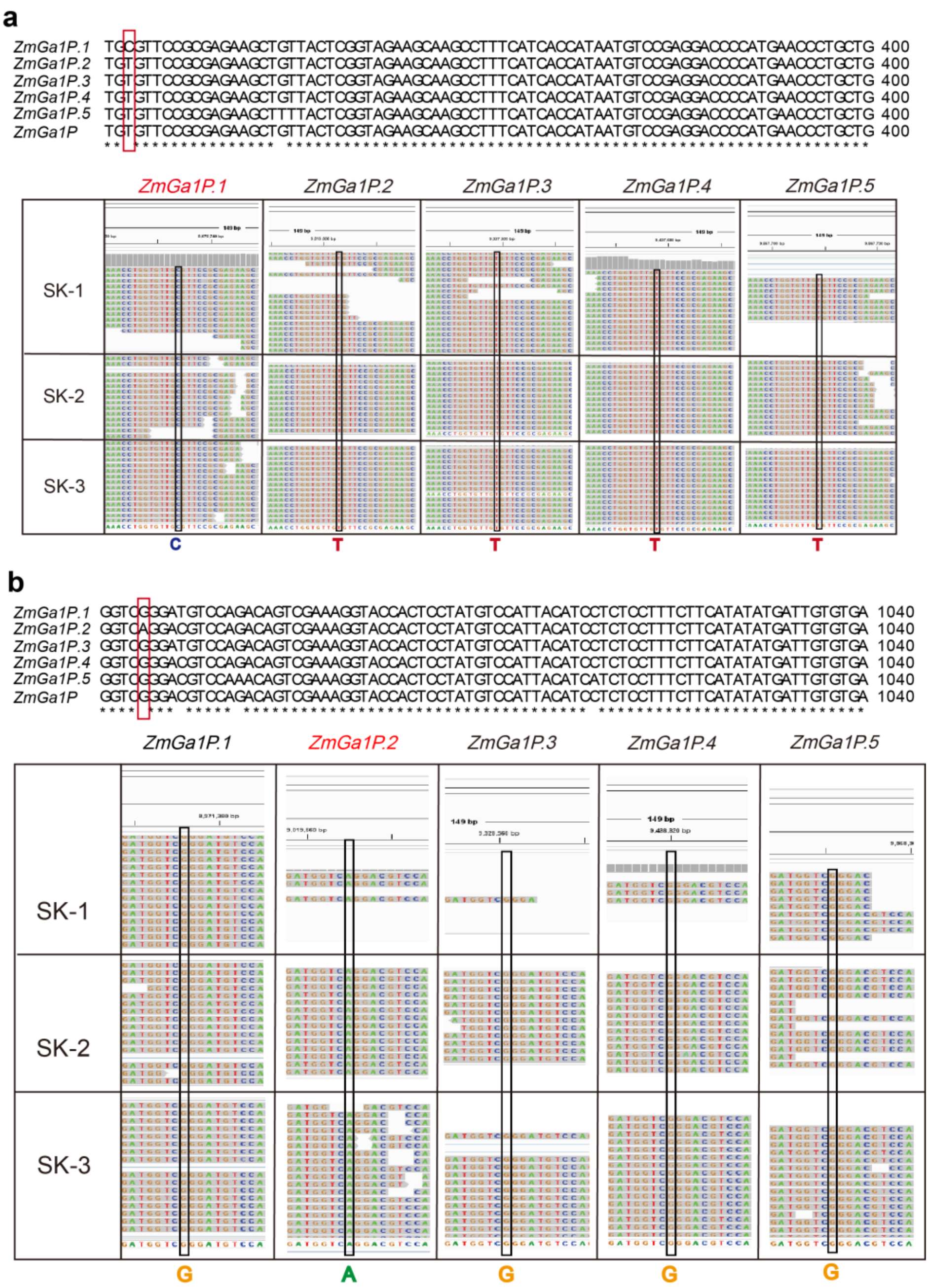

Supplementary Fig. 10. **SNPs in the RNA-seq data of SK pollen. (a)** Top, a unique SNP of *ZmGa1P.1* was detected in the SK genome. Bottom, the same SNP in RNA-seq data from SK pollen (with three replications). **(b)** Top, a unique SNP of *ZmGa1P.2* was detected in the SK genome. Bottom, the same SNP in RNA-seq data from SK pollen (with three replications).

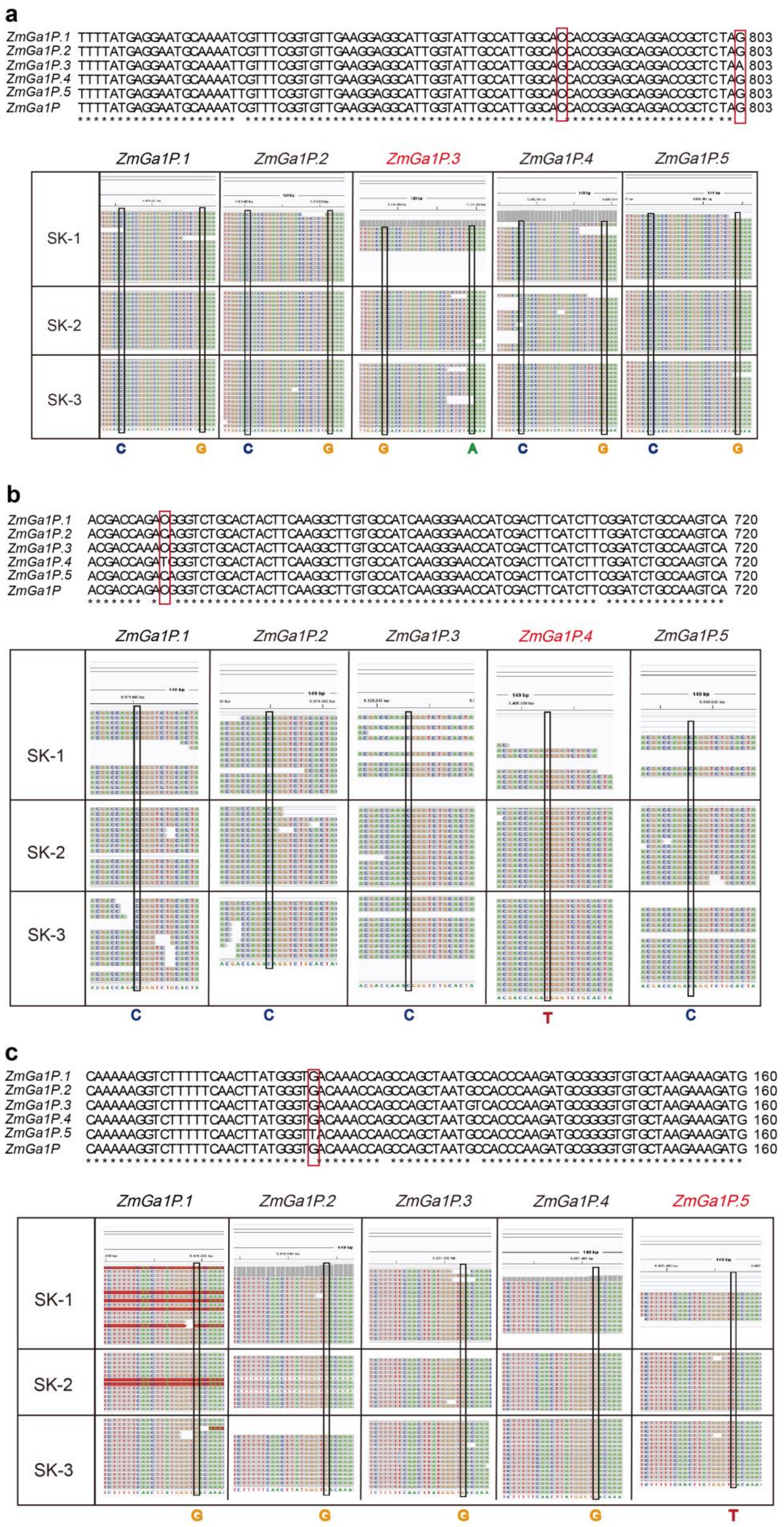
a
ZmGa1P.1 TTTTATGAGGAATGCAAAATCGTTTCGGTGTTGAAGGAGGCATTGGTATTGCCATTGGCACCACCGGAGCAGGACCGCTCTAG 803
ZmGa1P.2 TTTTATGAGGAATGCAAAATCGTTTCGGTGTTGAAGGAGGCATTGGTATTGCCATTGGCACCACCGGAGCAGGACCGCTCTAG 803
ZmGa1P.3 TTTTATGAGGAATGCAAAATTGTTTCGGTGTTGAAGGAGGCATTGGTATTGCCATTGGCAGCACCGGAGCAGGACCGCTCTAA 803
ZmGa1P.4 TTTTATGAGGAATGCAAAATCGTTTCGGTGTTGAAGGAGGCATTGGTATTGCCATTGGCACCACCGGAGCAGGACCGCTCTAG 803
ZmGa1P.5 TTTTATGAGGAATGCAAAATTGTTTCGGTGTTGAAGGAGGCATTGGTATTGCCATTGGCACCACCGGAGCAGGACCGCTCTAG 803
ZmGa1P TTTTATGAGGAATGCAAAATCGTTTCGGTGTTGAAGGAGGCATTGGTATTGCCATTGGCACCACCGGAGCAGGACCGCTCTAG 803
ZmGa1P.1
ZmGa1P.2
ZmGa1P.3
ZmGa1P.4
ZmGa1P.5
SK-1
SK-2
SK-3
C G C G G A C G C G
b
ZmGa1P.1 ACGACCAGACGGGTCTGCACTACTTCAAGGCTTGTGCCATCAAGGGAACCATCGACTTCATCTTCGGATCTGCCAAGTCA 720
ZmGa1P.2 ACGACCAGACAGGTCTGCACTACTTCAAGGCTTGTGCCATCAAGGGAACCATCGACTTCATCTTTGGATCTGCCAAGTCA 720
ZmGa1P.3 ACGACCAAACGGGTCTGCACTACTTCAAGGCTTGTGCCATCAAGGGAACCATCGACTTCATCTTCGGATCTGCCAAGTCA 720
ZmGa1P.4 ACGACCAGATGGGTCTGCACTACTTCAAGGCTTGTGCCATCAAGGGAACCATCGACTTCATCTTTGGATCTGCCAAGTCA 720
ZmGa1P.5 ACGACCAGACAGGTCTGCACTACTTCAAGGCTTGTGCCATCAAGGGAACCATCGACTTCATCTTCGGATCTGCCAAGTCA 720
ZmGa1P ACGACCAGACGGGTCTGCACTACTTCAAGGCTTGTGCCATCAAGGGAACCATCGACTTCATCTTCGGATCTGCCAAGTCA 720
ZmGa1P.1
ZmGa1P.2
ZmGa1P.3
ZmGa1P.4
ZmGa1P.5
SK-1
SK-2
SK-3
C C C T C
c
ZmGa1P.1 CAAAAAGGTCTTTTTCAACTTATGGGTGACAAACCAGCCAGCTAATGCCACCCAAGATGCGGGGTGTGCTAAGAAAGATG 160
ZmGa1P.2 CAAAAAGGTCTTTTTCAACTTATGGGTGACAAACCAGCCAGCTAATGCCACCCAAGATGCGGGGTGTGCTAAGAAAGATG 160
ZmGa1P.3 CAAAAAGGTCTTTTTCAACTTATGGGTGACAAACCAGCCAGCTAATGTCACCCAAGATGCGGGGTGTGCTAAGAAAGATG 160
ZmGa1P.4 CAAAAAGGTCTTTTTCAACTTATGGGTGACAAACCAGCCAGCTAATGCCACCCAAGATGCGGGGTGTGCTAAGAAAGATG 160
ZmGa1P.5 CAAAAAGGTCTTTTTCAACTTATGGGTTACAAACCAACCAGCTAATGCCACCCAAGATGCGGGGTGTGCTAAGAAAGATG 160
ZmGa1P CAAAAAGGTCTTTTTCAACTTATGGGTGACAAACCAGCCAGCTAATGCCACCCAAGATGCGGGGTGTGCTAAGAAAGATG 160
ZmGa1P.1
ZmGa1P.2
ZmGa1P.3
ZmGa1P.4
ZmGa1P.5
SK-1
SK-2
SK-3
G G G G T

Supplementary Fig. 11. **SNPs in the RNA-seq data of SK pollen. (a)** Top, a unique SNP of *ZmGa1P.3* was detected in the SK genome. Bottom, the same SNP in RNA-seq data from SK pollen (with three replications). **(b)** Top, a unique SNP of *ZmGa1P.4* was detected in the SK genome. Bottom, the same SNP in RNA-seq data from SK pollen (with three replications). **(c)** Top, a unique SNP of *ZmGa1P.5* was detected in the SK genome. Bottom, the same SNP in RNA-seq data from SK pollen (with three replications).

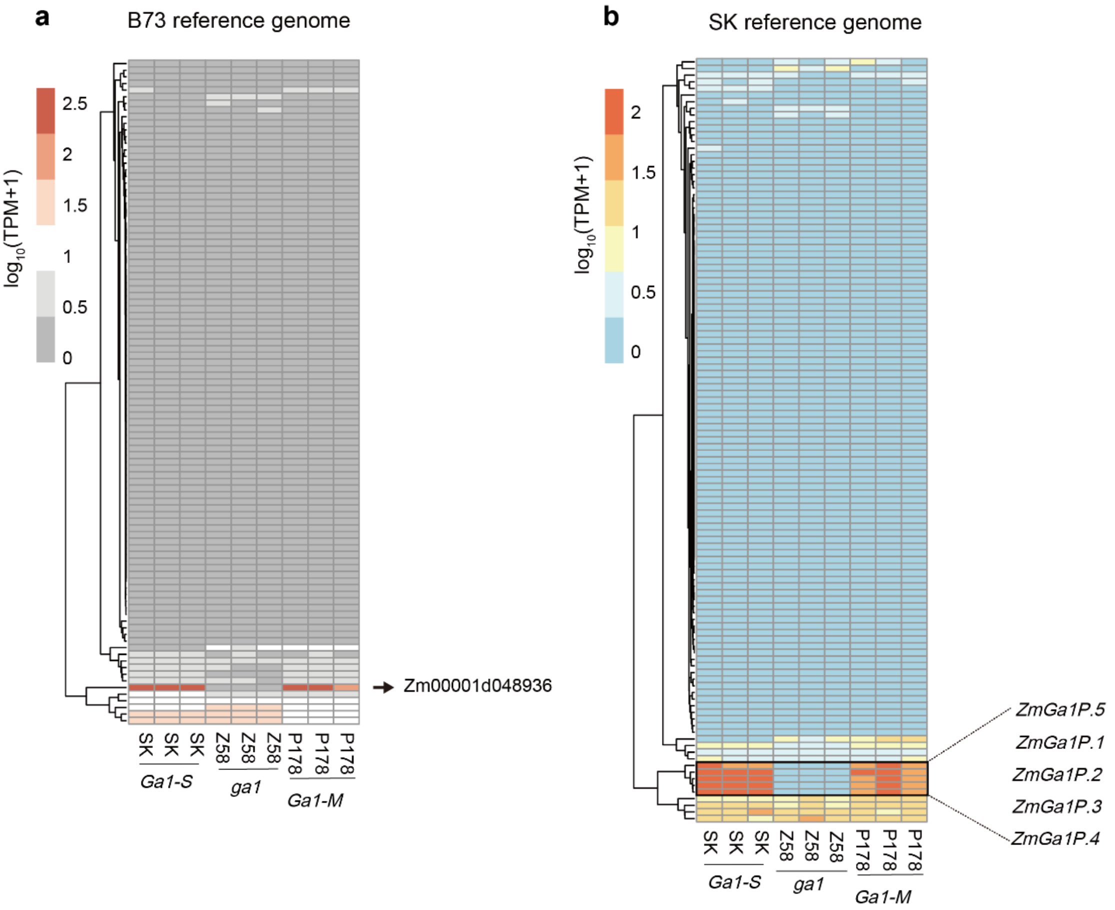

Supplementary Fig. 12. Expression analysis of genes in the *Ga1* locus in SK (*Ga1-S*), Z58 (*ga1*) and P178 (*Ga1-M*) pollen. (a) Evaluating expression level of the *Ga1* locus genes in pollen based on B73_v4 annotation information. TPM, Transcripts Per kilobase per Million reads. **(b)** Evaluating expression level of the *Ga1* locus genes in pollen based on SK annotation information. TPM, Transcripts Per kilobase per Million mapped reads.

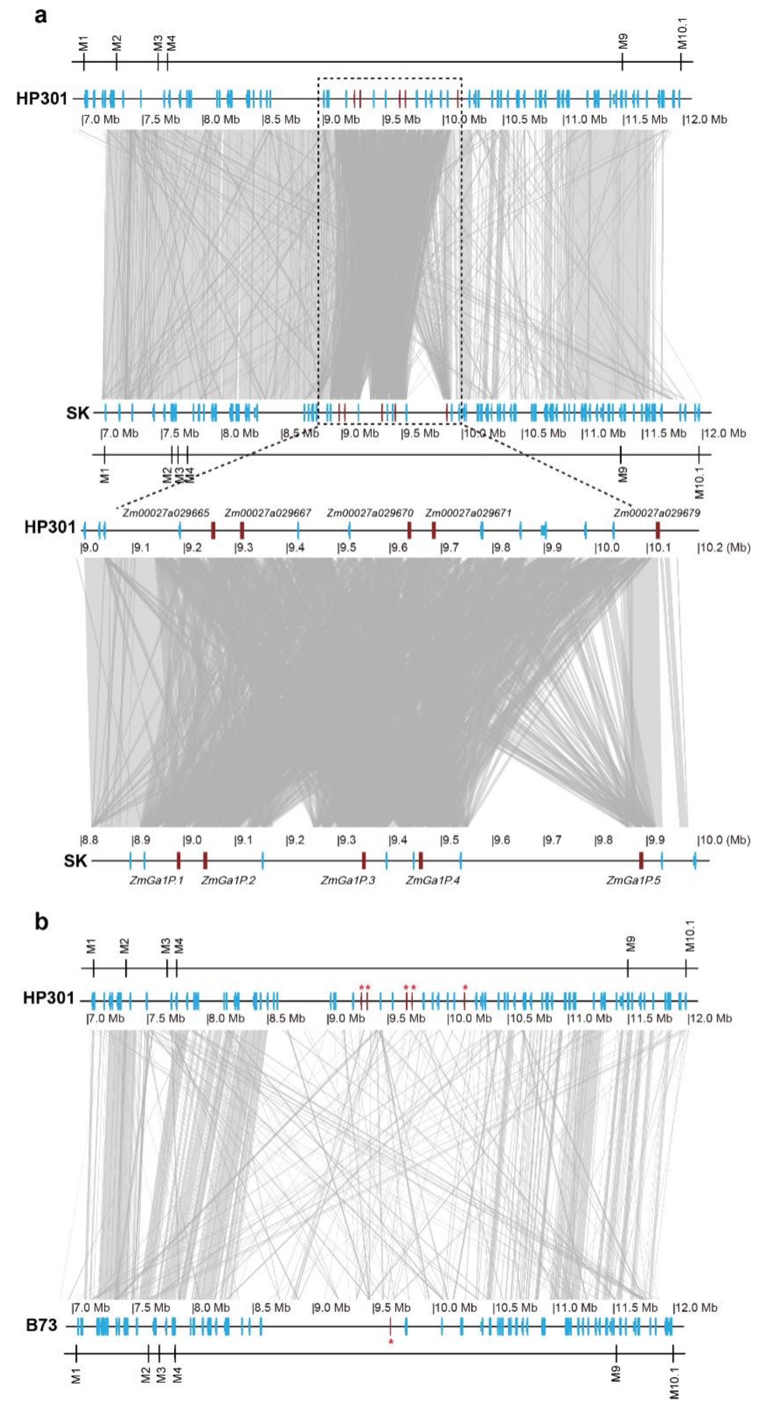

Supplementary Fig. 13. Genomic structure of the *Ga1* locus compared between SK, HP301 and B73 genomes. (a) Sequence alignment of the *Ga1* locus between HP301 and SK genomes. Synteny blocks are highlighted by gray lines. Annotated genes of the two genomes are highlighted in blue and red. Five *PME* genes of the SK genome and five homologous genes of the HP301 genome are highlighted in red. **(b)** Sequence alignment of the *Ga1* locus between HP301 and B73 genomes. Synteny blocks are highlighted by gray lines. Annotated genes of the two genomes are highlighted in blue and red. Red boxes and "*" highlighted in red represents five *PME* genes of the HP301 genome and one homologous gene on the B73 genome.

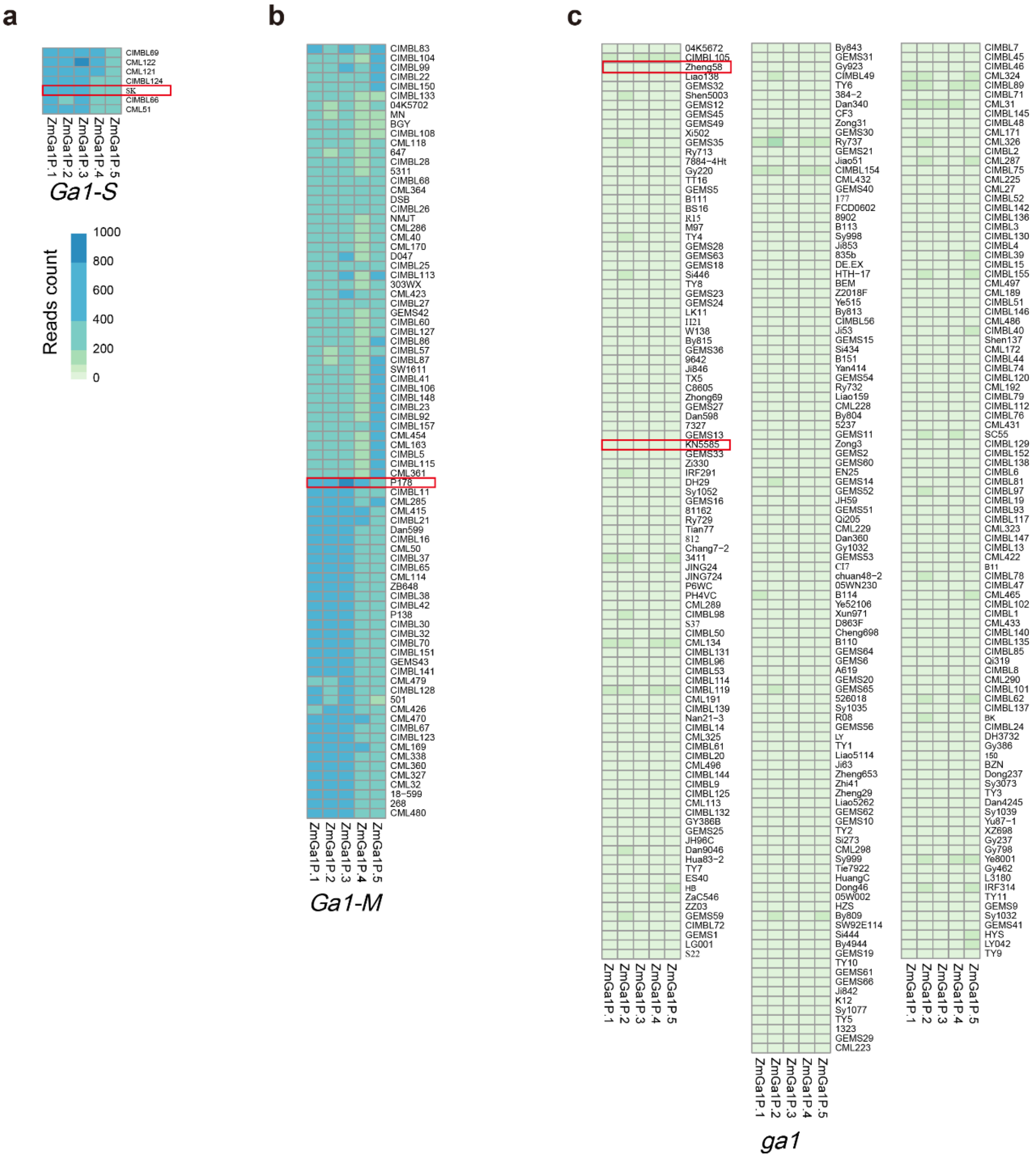

Supplementary Fig. 14. Mapped reads count of *ZmGa1Ps-m* genes in the AMP. (a) Mapped reads count of *ZmGa1Ps-m* genes in *Ga1-S* type lines. SK is highlighted with a red box. **(b)** Mapped reads count of *ZmGa1Ps-m* genes in *Ga1-M* type lines. P178 is highlighted with red boxes. **(c)** Mapped reads count of *ZmGa1Ps-m* genes in *ga1* type lines. Zheng58 and KN5585 are highlighted with red boxes.

a

b

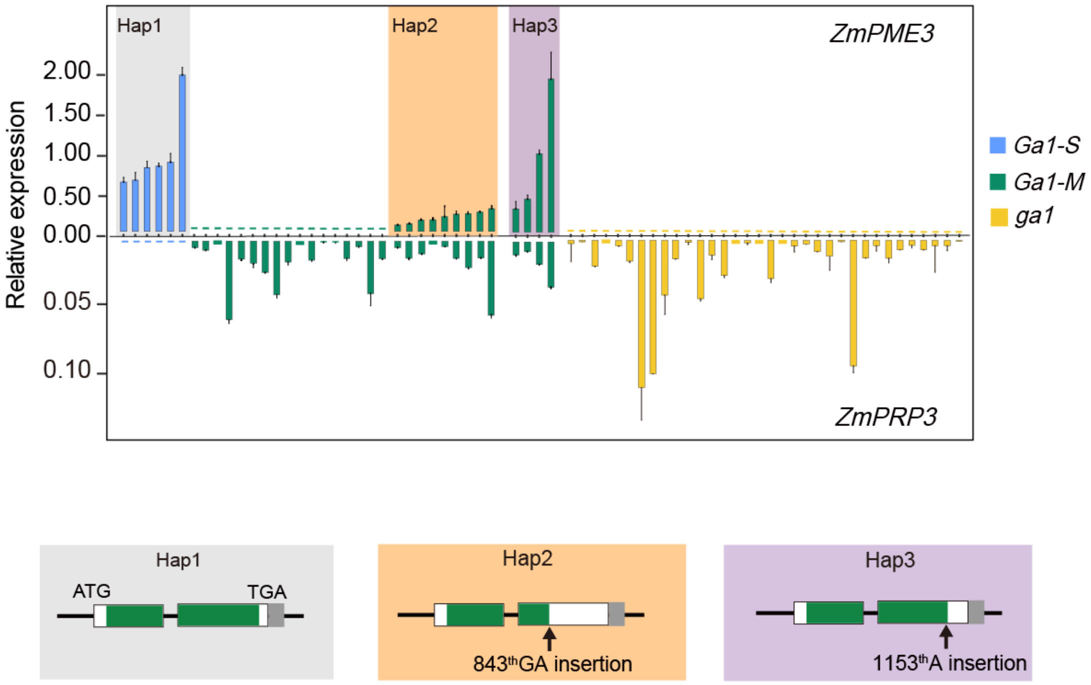

Supplementary Fig. 15. Evaluating the expression level of *ZmPME3* and *ZmPRP3* in silks of 70 inbred lines. (a) 70 silk samples of inbred lines from the AMP were used to evaluate the expression level of *ZmPME3* and *ZmPRP3* by quantitative RT-PCR analysis. Error bars represent mean + SD (n = 3). **(b)** Sequencing *ZmPME3* transcripts from *Ga1-M* and *Ga1-S* silks revealed three haplotypes in the AMP. The pectin methylesterase domains in *ZmPME3*^{SK} genes are highlight by green. White boxes represent coding sequence. UTR region is highlighted with grey boxes.

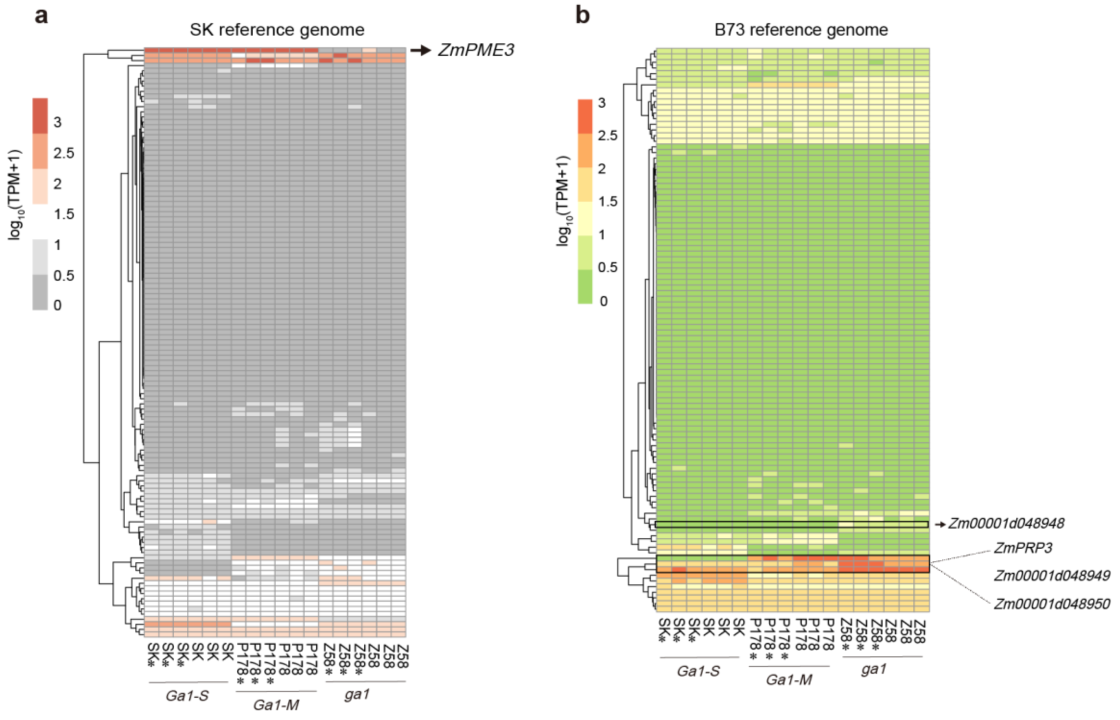

Supplementary Fig. 16. Expression analysis of genes in the *Ga1* locus in silks of SK (*Ga1-S)*, P178 *(Ga1-M)* and Z58 *(ga1)*. (a) Evaluating expression level of the *Ga1* locus genes in silks based on B73 v4 annotation information. "*" indicates non self-pollinated silk. TPM, Transcripts Per kilobase per Million mapped reads. **(b)** Evaluating expression level of the *Ga1* locus genes in silks based on SK annotation information. "*" indicates non self-pollinated silk. TPM, Transcripts Per kilobase per Million mapped reads.

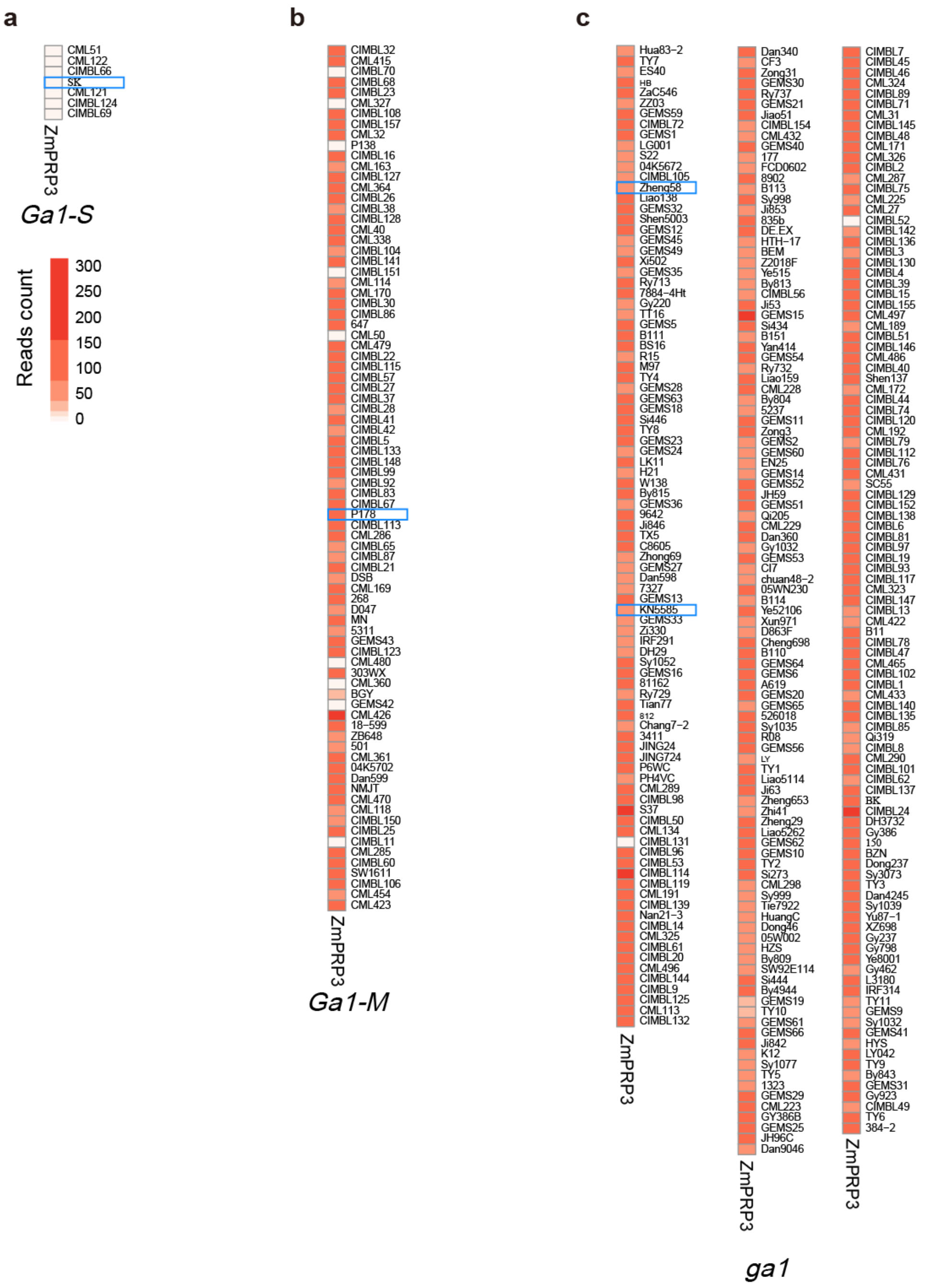

Supplementary Fig. 17. Mapped reads count of *ZmPRP3* in the AMP (a) Mapped reads count of *ZmPRP3* in *Ga1-S* type lines. SK is highlighted with a blue box. **(b)** Mapped reads count of *ZmPRP3* in *Ga1-M* type lines. P178 is highlighted with blue boxes. **(c)** Mapped reads count of *ZmPRP3* in *ga1* type lines. Zheng58 and KN5585 are highlighted with blue boxes.

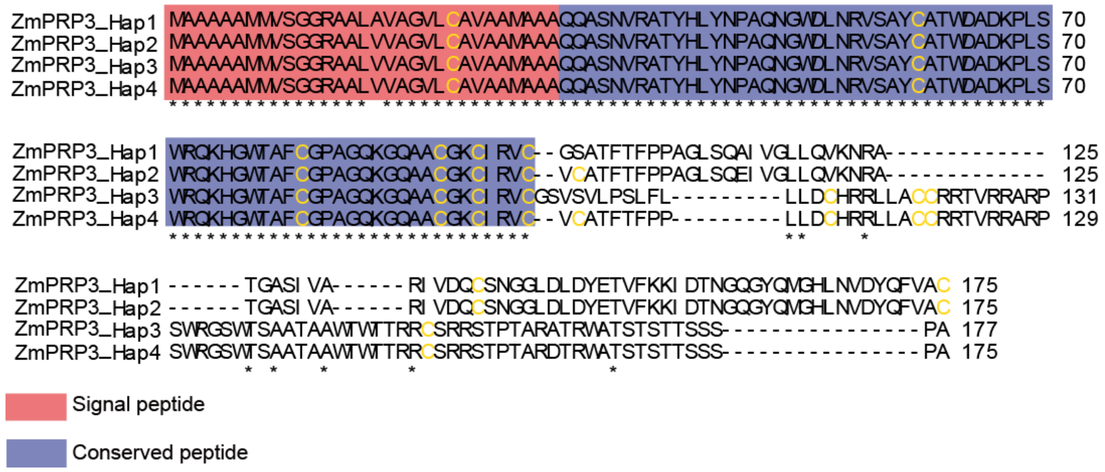

Supplementary Fig. 18. Protein sequence alignment of *ZmPRP3* from four haplotypes. The signal peptide is highlighted in red, the conserved peptide is highlighted in purple, and cysteines are drawn in yellow. “*” indicate identical amino acids.

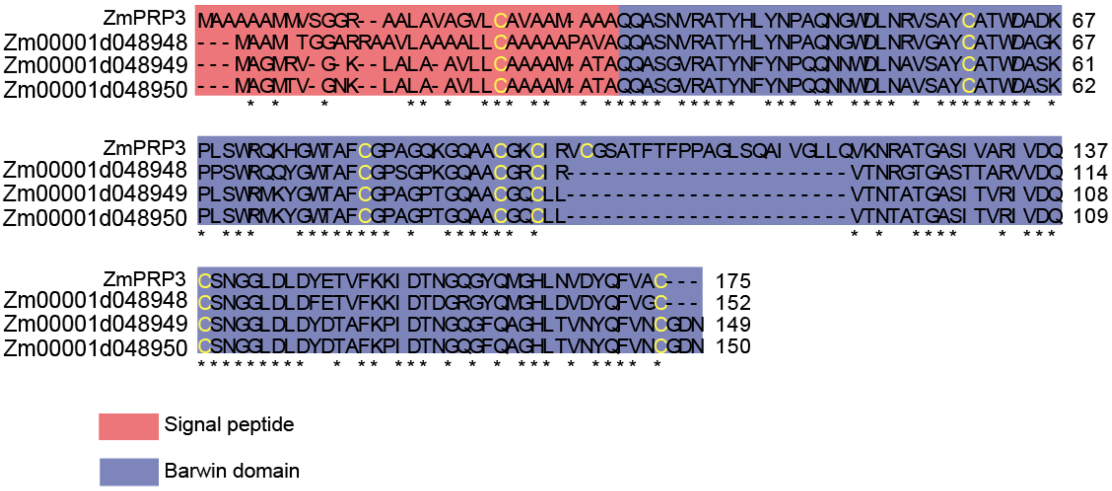

Supplementary Fig. 19. B73 protein sequence alignment of *ZmPRP3*, *Zm00001d048948, Zm00001d048949,* and *Zm00001d048950* at the *Ga1* locus. The signal peptide is highlighted in red, the Barwin domain is highlighted in purple, and cysteines are highlighted in yellow. "*" indicate identical amino acids.

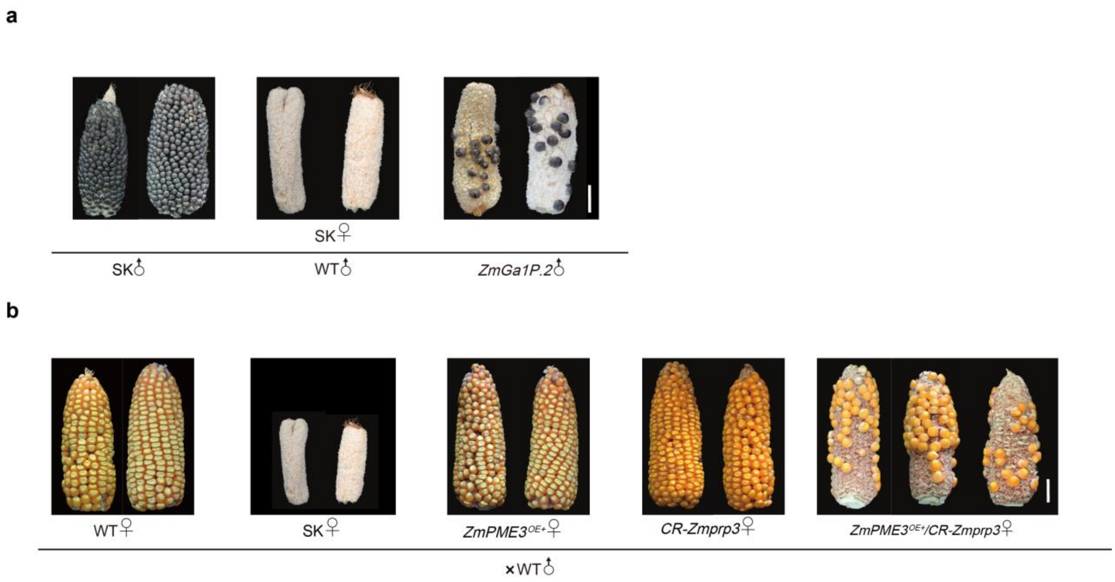

Supplementary Fig. 20. Crossing fertility test. (a) Crossing experiments showing ears of SK (*Ga1-S*) after self-pollination, pollinated by WT [KN5585 (*ga1*)] and transgenic plants pollen that over expressed *ZmGa1P.2.* Note that harvested kernel numbers were shown in Fig. 2c. Scale bar = 2 cm. **(b)** Crossing experiments showing ears of WT [(KN5585, *ga1*)] after self-pollination and ears of SK, *ZmPME3*$^{OE+}$, *CR-Zmprp3* and *ZmPME3*$^{OE+}$/ *CR-Zmprp3* after pollinated by WT pollen [KN5585 (*ga1*)]. Note that seed set ratios were shown in Fig. 3F. Scale bar = 2 cm.

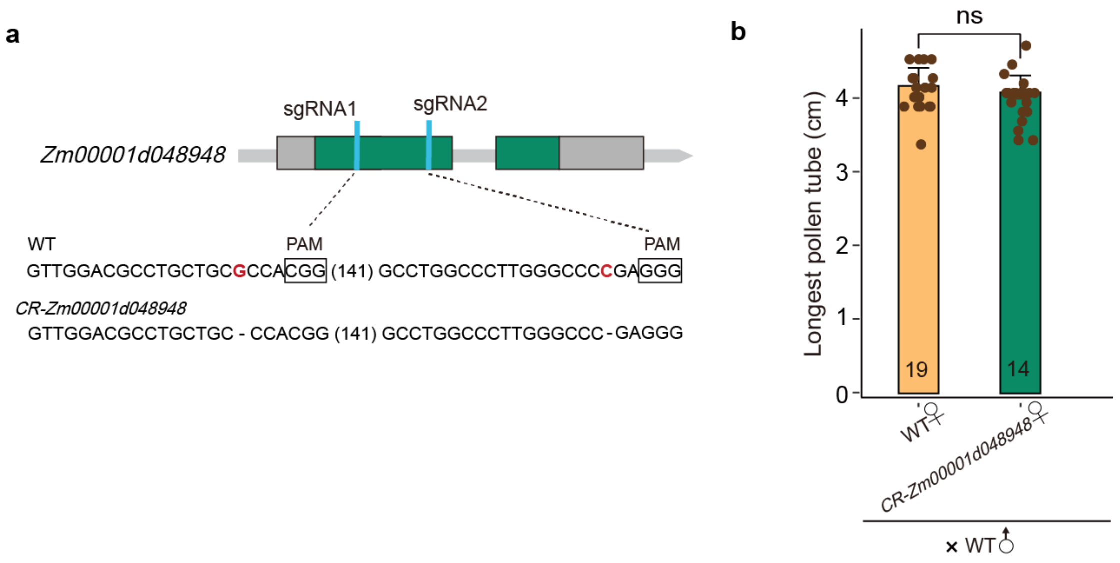

Supplementary Fig. 21. ***Zm00001d048948*** **has no effect on regulating pollen tube growth. (a)** Sequence of *Zm00001d048948* in CRISPR-Cas9 edited plants. Blue lines denote sgRNA, and the protospacer-adjacent motif (PAM, NGG) is indicated by black squares. The green boxes indicated the coding sequence, and UTR regions are highlighted with grey boxes. **(b)** Comparison of longest pollen tubes of WT [KN5585 (*ga1*)] in silks of WT and *Zm00001d048948* knock-out lines (*CR-Zm00001d048948)*. Error bars represent mean + SD, ns (not significant, P > 0.05, two-tailed Student's t-test). Numbers in each column indicate sample size. Source data are provided as a Source Data file.

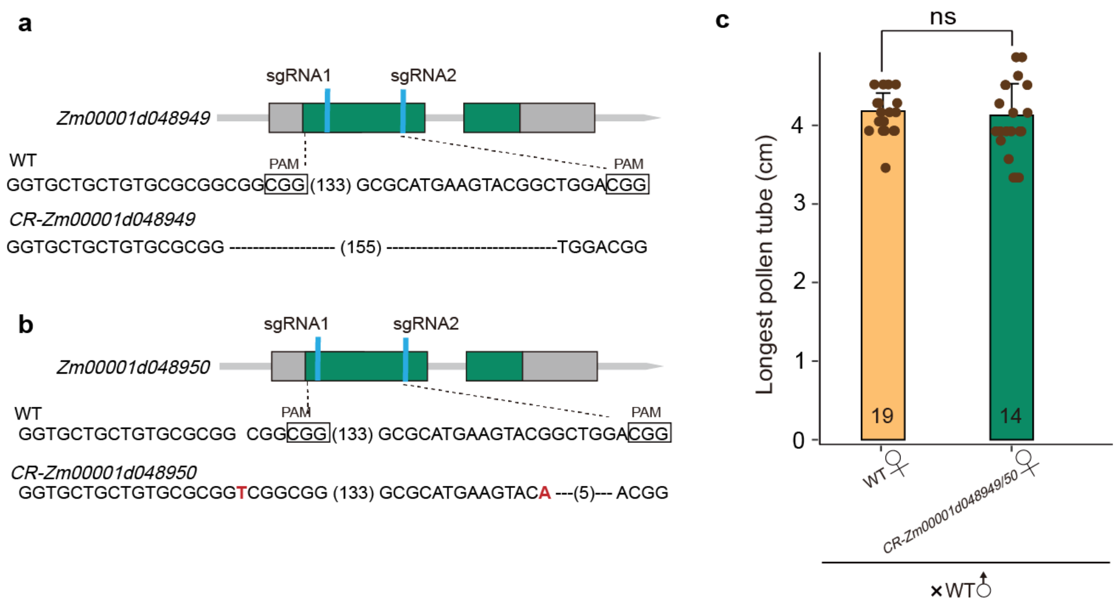

Supplementary Fig. 22. ***Zm00001d048949*** **and** ***Zm00001d048950*** **have no effect on regulating pollen tube growth.** **(a)** Sequence of *Zm00001d048949* in a CRISPR-Cas9 edited plant. Lines in blue denote sgRNA, and the protospacer-adjacent motif (PAM, NGG) is indicated by the black square. The green boxes indicate coding sequence, and UTR region is highlight with grey boxes. **(b)** Sequence of *Zm00001d048950* in a CRISPR-Cas9 edited plant. Lines in blue denote the sgRNA, and the protospacer-adjacent motif (PAM, NGG) is indicated by the black square**.** The green boxes indicated coding sequence, and UTR region is highlight with grey boxes. **(c)** Comparison of the longest pollen tubes of WT [KN5585 (*ga1*)] in the silks of WT and double mutants of *Zm00001d048949* and *Zm00001d048950 (CR-Zm00001d048949/50)*. Error bars represent mean + SD, ns (not significant, P > 0.05, two-tailed Student' s t-test). Numbers in each column indicate sample size. Source data are provided as a Source Data file.

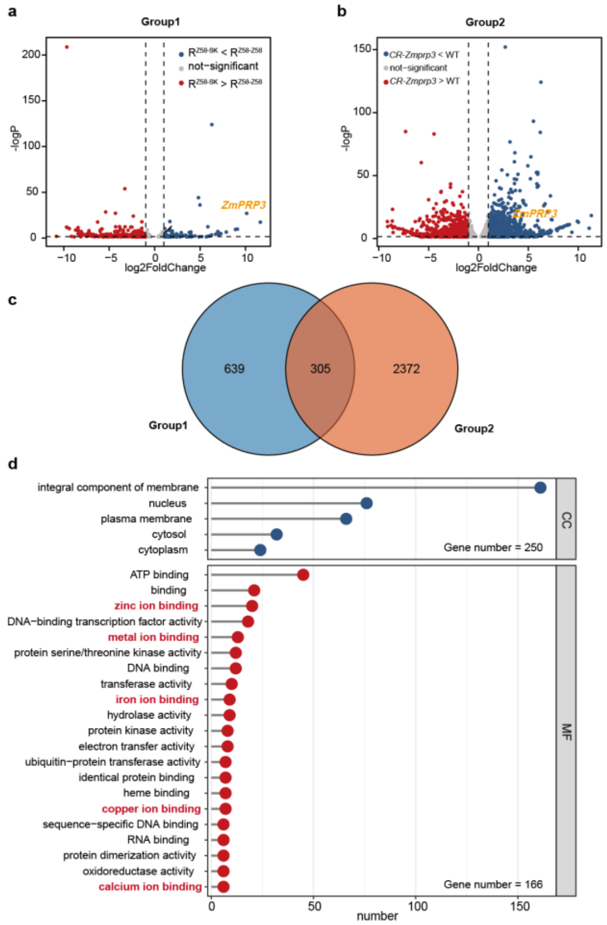

Supplementary Fig. 23. Differentially expressed genes (DEGs) in lines expressing and lacking *ZmPRP3* respectively. (a) Volcano plot showing DEGs in RL-Component1^{Z58}-Component1^{SK} (R$^{Z58\text{-}SK}$) and RL-Component1^{Z58}-Component1^{Z58} (R$^{Z58\text{-}Z58}$) silks. Two vertical lines indicate gene expression fold change (R$^{Z58\text{-}SK}$ vs. R$^{Z58\text{-}Z58}$) >2 and < −2, respectively, and the horizontal line indicates the adjusted *P* value (FDR q-value) of 0.05. *P* values were calculated by two-sided Wilcoxon rank-sum test. **(b)** Volcano plot showing DEGs in *ZmPRP3* knock-out lines and WT silks. Two vertical lines indicate gene expression fold change (*CR-Zmprp3* vs. WT) >2 and < −2, respectively, and the horizontal line indicates the adjusted *P* value (FDR q-value) of 0.01. *P* values were calculated by two-sided Wilcoxon rank-sum test. **(c)** Unique and shared DEGs between lines expressing and lacking *ZmPRP3*, respectively, are depicted in the 2-way Venn diagram. **(d)** List of DEGs that are enriched in five Cellular Component (CC) GO terms. More than half (n=166) of DEGs are associated with 20 Molecular Function (MF) GO terms. Molecular Function (MF) GO terms related to metal ion binding are highlighted in red. Source data are provided as a Source Data file.

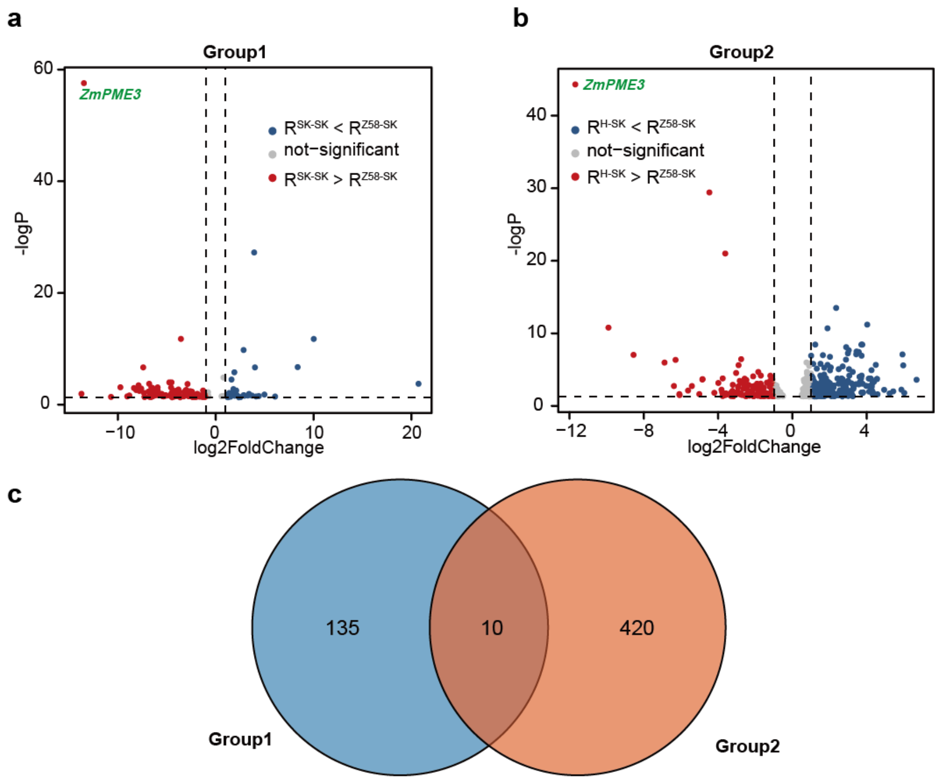

Supplementary Fig. 24. DEGs in lines expressing and lacking *ZmPME3*, respectively. (a) Volcano plot showing DEGs in RL-Component1^{SK}-Component1^{SK} (R^{SK-SK}) and RL-Component1^{Z58}-Component1^{SK} (R^{Z58-SK}) silks. Two vertical lines indicate gene expression fold change (R^{SK-SK} vs. R^{Z58-SK}) >2 and <−2, respectively, and the horizontal line indicates the adjusted *P* value (FDR q-value) of 0.05. *P* values were calculated by two-sided Wilcoxon rank-sum test. **(b)** Volcano plot showing DEGs between RL-Component1^{Hetrozygous}-Component1^{SK} (R^{H-SK}) and RL-Component1^{Z58}-Component1^{SK} (R^{Z58-SK}) silks. Two vertical lines indicate gene expression fold change (R^{H-SK} vs. R^{Z58-SK}) >2 and <−2, respectively, and the horizontal line indicates the adjusted *P* value (FDR q-value) of 0.01. *P* values were calculated by two-sided Wilcoxon rank-sum test. **(c)** Unique and shared DEGs between lines expressing and lacking *ZmPME3*, respectively, are depicted in the 2-way Venn diagram. Source data are provided as a Source Data file.

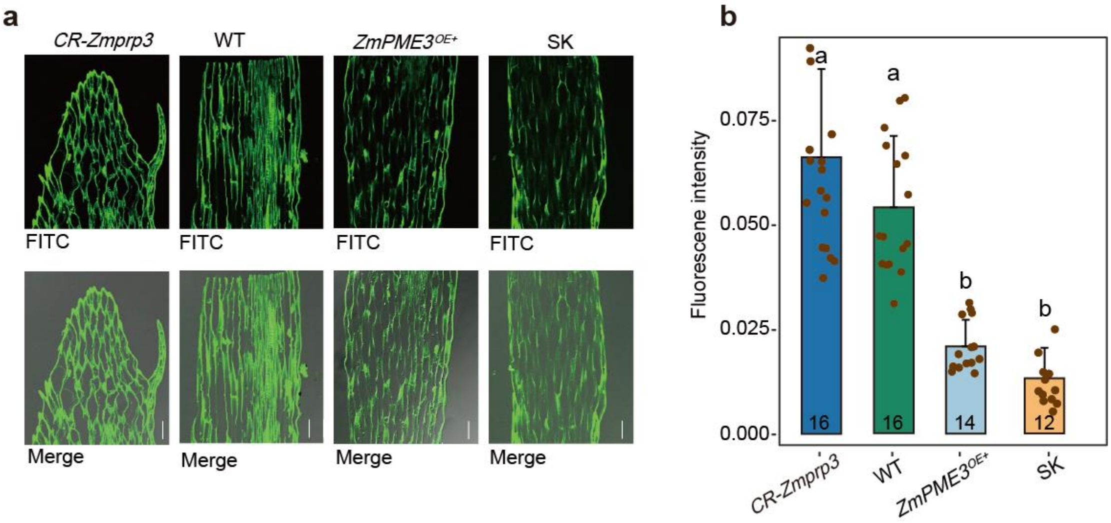

Supplementary Fig. 25. Immuno-detection of methylesterified pectin in silk cells. (a) Immuno-detection of methylesterified pectin in silk cells of SK (*Ga1-S*), WT [KN5585 (*ga1*)], *ZmPME3*$^{OE+}$ and *CR-Zmprp3* using the LM20 antibody. Scale bar = 50 µm, FITC, Fluorescein isothiocyanate. **(b)** Quantification of fluorescent signal intensity in silk cells shown in (a). Error bars represent mean + SD. a, b indicate that means differ according to the LSD test (P < 0.01). Numbers in each column indicate sample size. Source data are provided as a Source Data file.

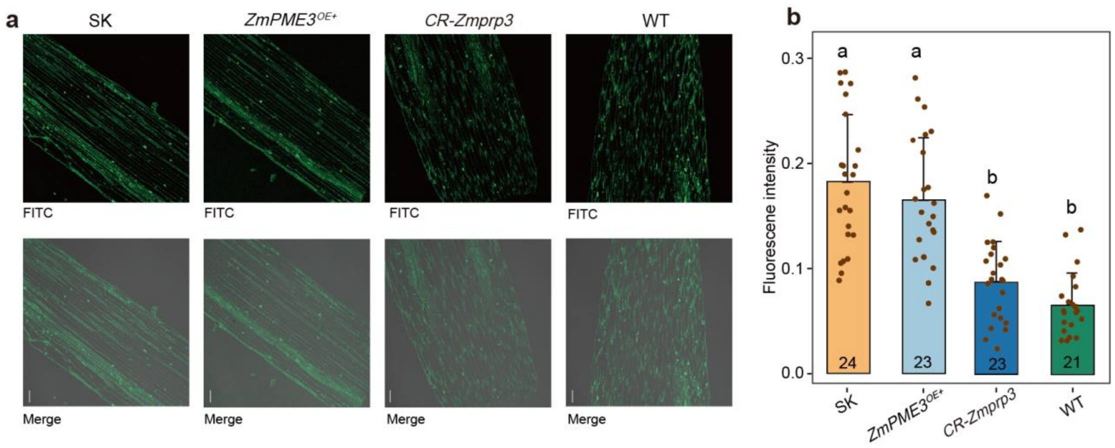

Supplementary Fig. 26. Immuno-detection of methylesterified pectin in silk cells. (a) Immuno-detection of methylesterified pectin in silk cells of SK (*Ga1-S*), WT [KN5585 (*ga1*)], *ZmPME3*$^{OE+}$ and *CR-Zmprp3* using the LM19 antibody. Scale bar = 50 μm, FITC, Fluorescein isothiocyanate. **(b)** Quantification of fluorescent signal intensity in silk cells shown in (a). Error bars represent mean + SD. a, b indicate that means differ according to the LSD test (P < 0.01). Numbers in each column indicate sample size. Source data are provided as a Source Data file.

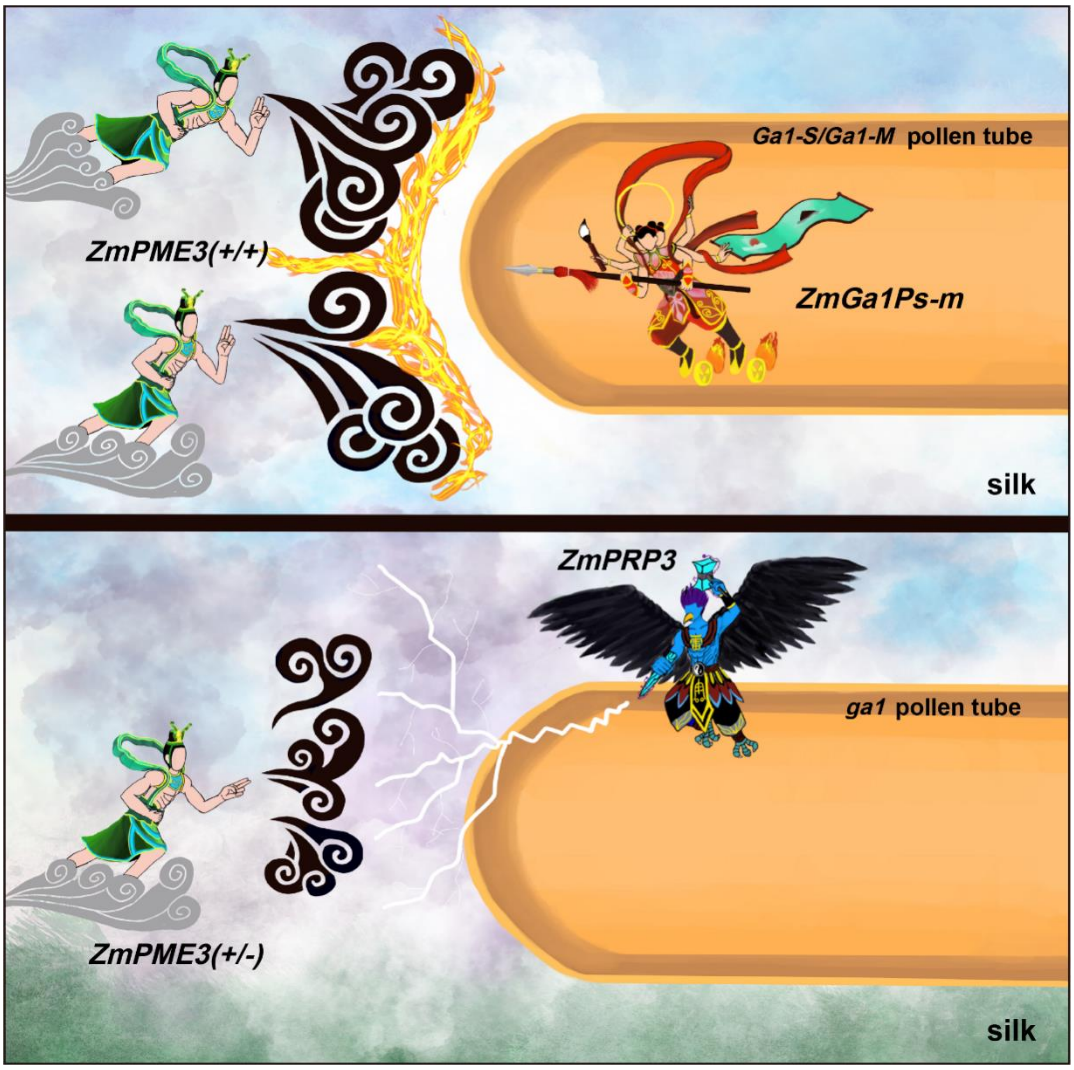

Supplementary Fig. 27. Genetic relationship between three types of genes of the *Ga1* locus. The characters from a popular Chinese mystery novel "the Legend of Deification" can be used to vividly illustrate the proposed three types of genes model for the UCI system. *ZmGa1Ps-m* genes are male determinants (as "Nezha", who has great magic power with three heads and six arms), which exist in *Ga1-S* and *Ga1-M* pollen, and can completely overcome cross incompatibility. Silk-expressed *ZmPME3* (as "Hmm-ha Two Generals", who are Hercules, and holding the sharp weapons) exists in *Ga1-S* silk, and is a female determinant that blocks *ga1* pollen tube grow through silk. Silk-expressed *ZmPRP3* (as "Leizhenzi", who is inferior to Nezha, but an excellent assist in the legend) acts as an accelerator, promoting the growth of *ga1* pollen tubes, when *ZmPME3* is heterozygous, introducing *ZmPRP3* can effectively break the *Ga1-S* barrier, promote *ga1* pollen tube growth.

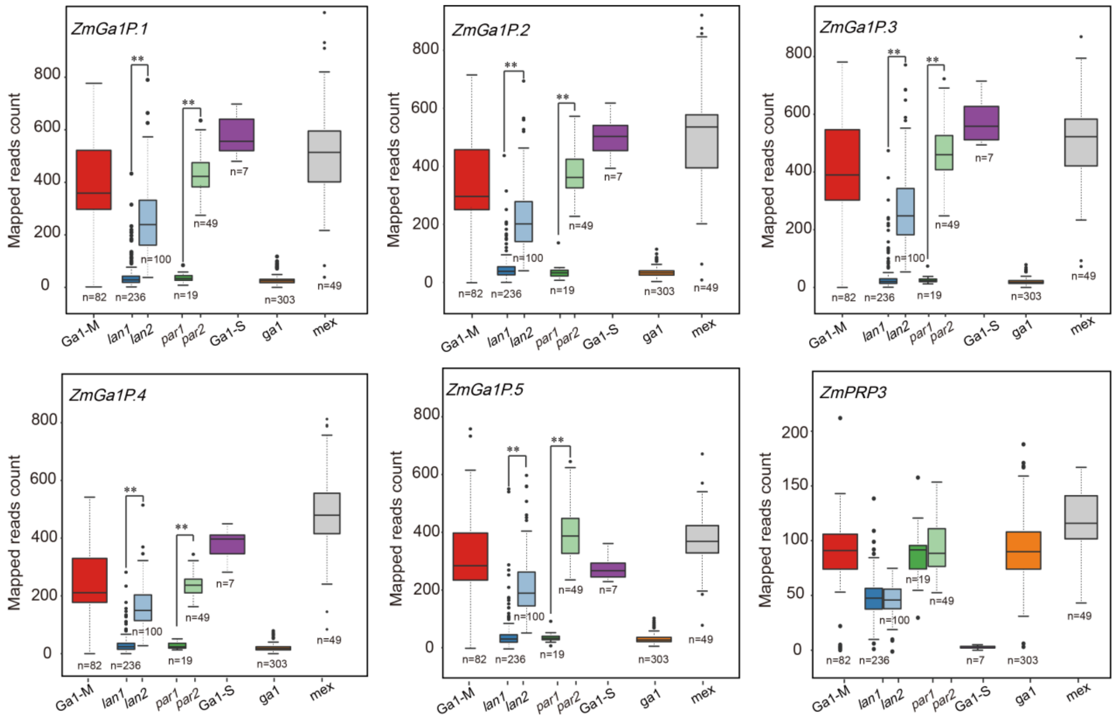

Supplementary Fig. 28. Genotyping *ZmGa1Ps-m* genes and *ZmPRP3* across cultivated, landrace and wild maize. Mapped reads count of *ZmGa1Ps-m* genes and *ZmPRP3* among two *parviglumis* sub-clusters *(par1, par2)*, *mexicana* (*mex*), two landrace sub-clusters (*lan1, lan2*) and *Ga1-S, Ga1-M* and *ga1* type lines. $^{**}P < 0.01$ (two-tailed Student' s t-test and Wilcoxon ranked sum non-parametric test). In box plots, the centre line represents the median, box edges delimit lower and upper quartiles and whiskers show the highest and lowest data points. n present sample size. Mapped reads counts are provided in Supplementary Data 6.

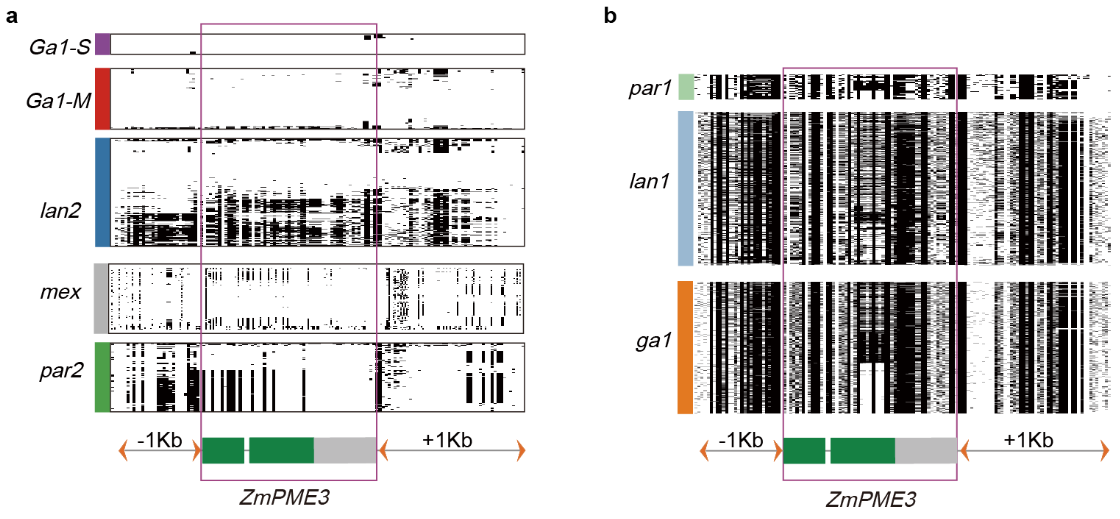

Supplementary Fig. 29. Local haplotype cluster of *ZmPME3*. (a) Constructing the local haplotype clusters of *ZmPME3* in *Ga1-S, Ga1-M*, *lan2, mexicana (mex)* and *par2* using snps from 1kb sequence upstream and downstream of *ZmPME3*. **(b)** Constructing the local haplotype clusters of *ZmPME3* in *ga1*, *lan1*, *par1* by using snps from 1kb sequence upstream and downstream of *ZmPME3*.

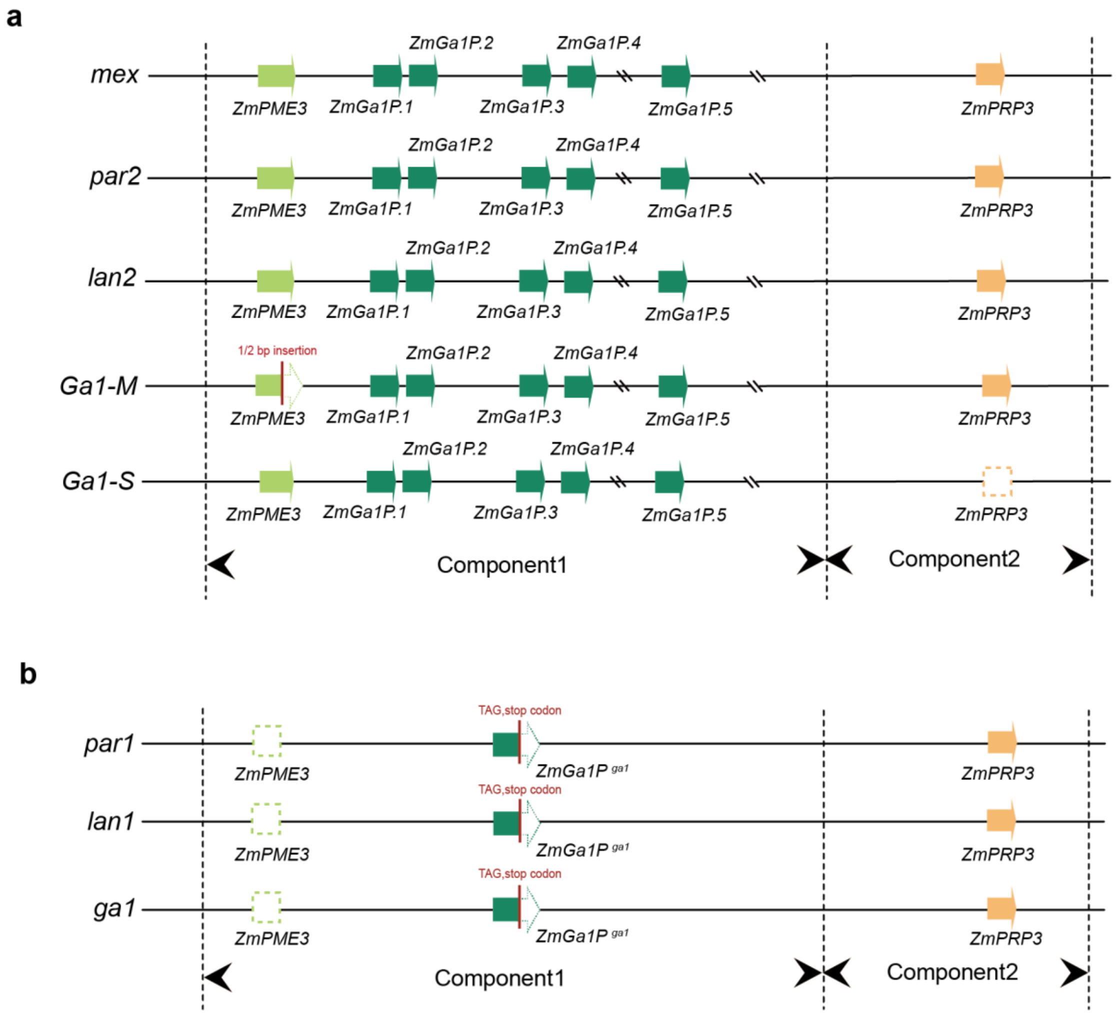

Supplementary Fig. 30. The sketch haplotype map of three types of genes of the *Ga1* locus on cultivated, landrace and wild maize genome. (a) *ZmPME3*, *ZmGa1Ps-m* and *ZmPRP3* on *par2*, *mexicana (mex)*, *lan2*, *Ga1-M* and *Ga1-S* type genome. Five *ZmGa1Ps-m* genes, one silk-expressed gene *ZmPME3*, and *ZmPRP3* are highlighted by dark green, light green and orange boxes. Absent or incomplete genes are indicated by dotted boxes. **(b)** *ZmPME3*, $ZmGa1P^{ga1}$ and *ZmPRP3* on *par1*, *lan1* and *ga1* type genome. $ZmGa1P^{ga1}$, *ZmPME3*, and *ZmPRP3* are highlighted by dark green, light green and orange boxes. Absent or incomplete genes are indicated by dotted boxes.
