# Supplementary material for: Three types of genes underlying the Gametophyte factor1 locus cause unilateral cross incompatibility in maize
Source: Nat Commun. 2022 Aug 3;13:4498. doi: 10.1038/s41467-022-32180-9 (PMC9349285; doi:10.1038/s41467-022-32180-9)
Supplement: Supplementary file 3 — Description of Additional Supplementary Files [file 41467_2022_32180_MOESM3_ESM.pdf]

Description of Additional Supplementary Files

File name: Supplementary Data 1.
Description: Annotated genes located in the *Ga1* ocus of SK genomes.

File name: Supplementary Data 2.
Description: Annotated genes located in the *Ga1* locus of B73 genomes.

File name: Supplementary Data 3.
Description: Expression level of annotated genes of the *Ga1* locus in pollen.

File name: Supplementary Data 4.
Description: Expression level of annotated genes of the *Ga1* locus in silk.

File name: Supplementary Data 5.
Description: 70 maize inbred lines from the AMP used for quantitative RT-PCR of *ZmPME3* and *ZmPRP3*.

File name: Supplementary Data 6.
Description: Mapped reads count of *ZmGa1Ps-m* genes, *ZmPME3* and *ZmPRP3* in *parviglumis*, *mexicana*, landraces and the AMP.

File name: Supplementary Data 7.
Description: Test results of Shapiro-Wilk normality test.
