# Supplementary material for: Three types of genes underlying the Gametophyte factor1 locus cause unilateral cross incompatibility in maize
Source: Nat Commun. 2022 Aug 3;13:4498. doi: 10.1038/s41467-022-32180-9 (PMC9349285; doi:10.1038/s41467-022-32180-9)
Supplement: Supplementary file 11 — Reporting Summary [file 41467_2022_32180_MOESM11_ESM.pdf]

nature portfolio

Corresponding author(s): Jianbing Yan

Last updated by author(s): YYYY-MM-DD

Reporting Summary

Nature Portfolio wishes to improve the reproducibility of the work that we publish. This form provides structure for consistency and transparency in reporting. For further information on Nature Portfolio policies, see our Editorial Policies and the Editorial Policy Checklist.

Statistics

For all statistical analyses, confirm that the following items are present in the figure legend, table legend, main text, or Methods section.

n/a	Confirmed	
☐	☒	The exact sample size (*n*) for each experimental group/condition, given as a discrete number and unit of measurement
☐	☒	A statement on whether measurements were taken from distinct samples or whether the same sample was measured repeatedly
☐	☒	The statistical test(s) used AND whether they are one- or two-sided<br>*Only common tests should be described solely by name; describe more complex techniques in the Methods section.*
☒	☐	A description of all covariates tested
☐	☒	A description of any assumptions or corrections, such as tests of normality and adjustment for multiple comparisons
☐	☒	A full description of the statistical parameters including central tendency (e.g. means) or other basic estimates (e.g. regression coefficient) AND variation (e.g. standard deviation) or associated estimates of uncertainty (e.g. confidence intervals)
☐	☒	For null hypothesis testing, the test statistic (e.g. *F*, *t*, *r*) with confidence intervals, effect sizes, degrees of freedom and *P* value noted<br>*Give P values as exact values whenever suitable.*
☒	☐	For Bayesian analysis, information on the choice of priors and Markov chain Monte Carlo settings
☒	☐	For hierarchical and complex designs, identification of the appropriate level for tests and full reporting of outcomes
☒	☐	Estimates of effect sizes (e.g. Cohen's *d*, Pearson's *r*), indicating how they were calculated

Our web collection on statistics for biologists contains articles on many of the points above.

Software and code

Policy information about availability of computer code

Data collection	All sequencing library were prepared in house and raw reads were generated on illumina sequencing platform with manufacture's instruction. An expanded RNA-seq for pollen and silk (PRJNA778568) and 340 maize landrace re-sequencing data (PRJNA783885) are public data.
Data analysis	Software used included : Trimmomatic, Bowtie2, BLAST local, GATK, TASSEL 3.0, RSEM-1.3.0, mafft-7.037, subread-1.6.3 tools featureCounts, Samtools, Picard, Smartpca, ImageJ, as well as R-3.4.0. Detailed parameters of each of the programs are mentioned in relevant sections in Methods.

For manuscripts utilizing custom algorithms or software that are central to the research but not yet described in published literature, software must be made available to editors and reviewers. We strongly encourage code deposition in a community repository (e.g. GitHub). See the Nature Portfolio guidelines for submitting code & software for further information.

Data

Policy information about availability of data

All manuscripts must include a data availability statement. This statement should provide the following information, where applicable:

- Accession codes, unique identifiers, or web links for publicly available datasets
- A description of any restrictions on data availability
- For clinical datasets or third party data, please ensure that the statement adheres to our policy

RNA-seq raw data of pollen and silk has been uploaded to NCBI SRA with BioProject accession number : PRJNA778568. Landrace re-sequencing data has been uploaded to NCBI SRA with BioProject accession number : PRJNA783885

Field-specific reporting

Please select the one below that is the best fit for your research. If you are not sure, read the appropriate sections before making your selection.

☒ Life sciences ☐ Behavioural & social sciences ☐ Ecological, evolutionary & environmental sciences

For a reference copy of the document with all sections, see nature.com/documents/nr-reporting-summary-flat.pdf

Life sciences study design

All studies must disclose on these points even when the disclosure is negative.

Sample size	No sample size is calculated. The sample size is the number of inbred lines of population.
Data exclusions	During principal component analysis, those variants with minor allele frequency (MAF) less than 5% and missing rate > 70% were excluded. This is common applied since the current method has no power to uncover this kind of rare alleles.
Replication	All experimental data was reliably reproduced in multiple independent experiments as indicated in the figure legends
Randomization	The sample of re-sequencing ZmPRP3 and ZmPME3 were randomly collected with 70 individuals from maize association mapping panel.
Blinding	No blinding was used

Reporting for specific materials, systems and methods

We require information from authors about some types of materials, experimental systems and methods used in many studies. Here, indicate whether each material, system or method listed is relevant to your study. If you are not sure if a list item applies to your research, read the appropriate section before selecting a response.

Materials & experimental systems

n/a	Involved in the study
☐	☒ Antibodies
☒	☐ Eukaryotic cell lines
☒	☐ Palaeontology and archaeology
☒	☐ Animals and other organisms
☒	☐ Human research participants
☒	☐ Clinical data
☒	☐ Dual use research of concern

Methods

n/a	Involved in the study
☒	☐ ChIP-seq
☒	☐ Flow cytometry
☒	☐ MRI-based neuroimaging

Antibodies

Antibodies used	Antibody : LM20 (plant probes) , LM19(plant probes), Anti-rat IgG (ABclonal) used for immunolabeling analysis
Validation	Antibody informations see below:<br>LM20: http://glycomics.ccrc.uga.edu/wall2/jsp/abdetails.jsp?abnumber=199&abname=LM20<br>LM19:http://glycomics.ccrc.uga.edu/wall2/jsp/abdetails.jsp?abname=LM19<br>Anti-rat IgG : https://abclonal.com.cn/search/index?catid=56&keyword=Anti-ra<br>+IgG&__hash__=6666cd76f96956469e7be39d750cc7d9_10a2d5b015028d6ea631308281f13a12&Searchbar=
